# One-Pot Synthesis of Carvyl Acetate from α‑Pinene Oxide under Catalysis by Zeolite H‑Beta 25

**DOI:** 10.1021/acsomega.5c12898

**Published:** 2026-03-19

**Authors:** Anna Rejzková, Marek Plachý, Eliška Vyskočilová

**Affiliations:** Department of Organic Technology, 52735 University of Chemistry and Technology, Prague, Technická 5 166 28 Prague 6, Czech Republic

## Abstract

The one-pot synthesis of carvyl acetate from α-pinene oxide was systematically investigated with respect to solvent selection, reaction temperature, catalyst loading, reactant molar ratios, and solvent volume to optimize the reaction conditions. The highest yield of carvyl acetate (47% after 4 h) was obtained using 20 wt % H-Beta 25 zeolite at 90 °C, with a molar ratio of α-pinene oxide:acetic anhydride:*N,N*-dimethylformamide of 1:8:8. The scalability of the process and the reusability of the catalyst were also evaluated. The catalyst demonstrated good stability and could be regenerated by calcination for repeated use. Kinetic parameters, including reaction rate constants and activation energy, were determined to provide deeper mechanistic insight. The results suggest that this method offers a robust and potentially scalable route for the industrial production of carvyl acetate from α-pinene oxide.

## Graphical abstract

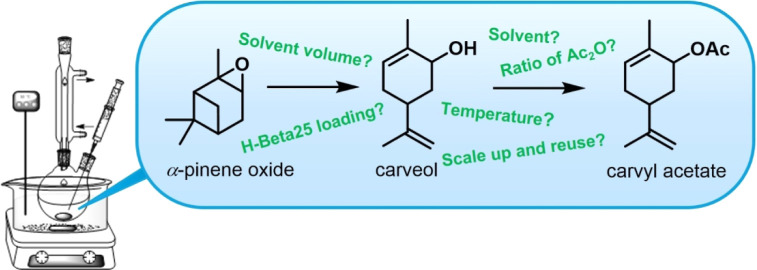

## Introduction

Carvyl acetate (2-methyl-5-(prop-1-en-2-yl)­cyclohex-2-en-1-yl acetate) is a colorless, oily terpene derivative naturally present in essential oils of plants such as mint, caraway, and celery. Its sweet, minty aroma makes it valuable in the fragrance and food industries.^1,2^ In addition to its sensory properties, carvyl acetate exhibits antibacterial activity against *Streptococcus mutans*, a major contributor to dental caries.^3^ Related carveol esters have also been shown to enhance the transdermal delivery of 5-fluorouracil while maintaining favorable stability.^4^

Due to its low natural abundance, carvyl acetate must be produced synthetically, typically via the acylation of terpenic alcohols. One of the most common methods involves the reaction of the corresponding alcohol with an acylating agent, such as a carboxylic acid, acid halide, or anhydride. This transformation is usually catalyzed by Brønsted acids (e.g., HNO_3_), Lewis acids (e.g., Fe­(NO_3_)_3_ ^5^), bases, or amines.^6^

When acetic anhydride was used as the acylating agent, basic conditions with amine catalysts—such as pyridine, triethylamine, or tri-*n*-octylamine—were employed.^7^ Carvyl acetate was obtained with a yield of 86% and purity of 74%. An example of acid-catalyzed acylation of a cyclic terpenic alcohol was the reaction of perillyl alcohol with acetic anhydride to form perillyl acetate, using Zn­(ClO_4_)_2_·6H_2_O. Under mild conditions, a 90% yield of perillyl acetate was achieved.^8^

Although terpenic alcohols are important intermediates in the synthesis of esters, such as carvyl acetate, they are often less accessible than their parent terpenes or epoxidized derivatives. Acid-catalyzed isomerization of α-pinene oxide offers a promising route to carveol, though achieving high selectivity remains challenging due to the formation of multiple side products.^9^

Brønsted-acid catalysts have proven effective for the isomerization of α-pinene oxide to carveol.^7–9^ Among zeolite-based systems, H-Beta 25 ^10^ showed the highest initial reaction rate due to its strong Brønsted acidity. The highest carveol selectivity—42% and 43%—was achieved with H-Beta-300 and Fe-Beta-300, respectively, after 3 h in DMA at 140 °C with 25 wt % catalyst loading.^10^

Sol–gel-derived Sn/SiO_2_ and Ce/SiO_2_ catalysts^11^ also showed promising results, reaching 73% carveol selectivity at 98% conversion (DMA, 140 °C, 33 wt %). A heteropolyacid H_3_PW_12_O_40_ on silica gave 29% carveol selectivity at 15 °C (0.6 wt %), though campholenic aldehyde was the main product despite the strong acidity.^12^

Montmorillonite, a layered phyllosilicate, is also a promising catalyst due to its low cost and availability. When used in cyclohexane at 30 °C, it afforded 13–15% carveol selectivity at nearly complete conversion of α-pinene oxide.^13^

The choice of solvent plays a crucial role in the isomerization of α-pinene oxide. In nonpolar solvents such as toluene or cyclohexane, campholenic aldehyde is typically the main product. In contrast, basic solvents like *N*,*N-*dimethylacetamide (DMA) or *N*,*N-*dimethylformamide (DMF) favor the formation of carveol.^14–16^

Direct acylation of terpenic epoxides has remained relatively unexplored. Štekrová et al.^17^ investigated the synthesis of perillyl acetate from β-pinene oxide using both heterogeneous (e.g., montmorillonite K-10, USY zeolite) and homogeneous acid catalysts.

The direct synthesis of carvyl acetate from α-pinene oxide using acetic anhydride was investigated in a study.^18^ Under specific conditions (DCM, 20 °C, 166.7 wt % montmorillonite), carvyl acetate was obtained in a 2% isolated yield after 8 h. The study^18^ also evaluated beta zeolite as an alternative catalyst.

In response to the growing interest in biomass-derived compounds and sustainable synthetic methodologies, the catalytic upgrading of α-pinene oxide into value-added products represents a promising research avenue. In this study, we investigate the catalytic performance of zeolite beta in the one-pot synthesis of carvyl acetate directly from α-pinene oxide (Figure 1). The central objective is to elucidate whether the transformation proceeds via a sequential pathway involving carveol as a reaction intermediate or whether a direct conversion can be achieved under the optimized reaction conditions. Particular emphasis is placed on the role of the solvent, reaction temperature, catalyst loading, and reactant ratios in enabling both the isomerization and acetylation steps within a single reaction system. Kinetic parameters are evaluated to provide mechanistic insight into the reaction pathway. This approach aims to establish a sustainable and efficient synthetic route to carvyl acetate from renewable α-pinene oxide while avoiding the isolation of carveol, which is challenging due to its similar boiling point to campholenic aldehyde, a major byproduct formed during α-pinene oxide isomerization.

**Figure 1:**
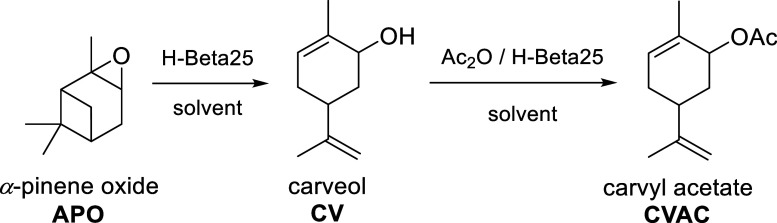
Scheme of one-pot synthesis of carvyl acetate.

## Experimental Section

All chemicals were purchased from commercial suppliers and used without any purification, unless stated otherwise. α-Pinene oxide (≥95%) was obtained from the Tokyo Chemical Industry. Acetic anhydride (p.a.), acetonitrile (p.a.), and toluene (p.a.) were purchased from Penta. Dimethyl sulfoxide (DMSO, p.a.) and *N,N*-dimethylformamide (DMF, ≥99.5%) were purchased from Lach-Ner. *N,N*-Dimethylacetamide (DMAc, ≥99%) was purchased from Merck together with carveol (≥99%) and tetramethylurea (≥99%). Zeolite beta (Si/Al = 25) was obtained from Zeolyst Int. All solvents were of analytical grade.

### Reaction Procedure

The one-pot production of carvyl acetate from α-pinene oxide was carried out in a round-bottom flask with a side neck and septum, equipped with a magnetic stirrer, and heated by a temperature-controlled oil bath and with a reflux condenser cooled by water. In a typical experiment, α-pinene oxide (1 g), acetic anhydride (typically 5.365 g in a ratio of 1:8), and solvent (typically 4 mL) were mixed in the desired molar ratio and preheated to the target temperature (50–110 °C) under intensive stirring in an oil bath. After a tempering period of 10 min at the given temperature, the catalyst (5–40 wt % initial mass of α-pinene oxide) was added, thereby initiating the reaction. Aliquots of the reaction mixture (typically 0.2 mL) were withdrawn at regular time intervals using a syringe through the side neck with a septum, after which the catalyst was separated by centrifugation. The liquid phase of the reaction mixture was then diluted with 1 mL of toluene and subsequently analyzed by gas chromatography. The experimental error was established as 5% by repeating of the reaction at the same conditions.

The optimization process was conducted by systematically varying a single experimental parameter (e.g., temperature) while maintaining all other reaction conditions constant. This approach enabled the isolated assessment of the specific effect of the selected variable on the reaction outcome. The logical sequence of the studied effect was dependent on the findings from the previous effect.

### Catalyst Manipulation

Zeolite beta (Si/Al = 25) as an ammonium form was calcined in air at 500 °C for 3 h prior to use for achieving hydrogen from and subsequently stored in a desiccator to maintain its dryness. After the chosen reaction cycle, the catalyst was separated by filtration, washed thoroughly with toluene, dried at 100 °C for 12 h, and calcined at 500 °C for 3 h before reuse. The recovered catalyst was then used in the next cycle under identical reaction conditions.

### Product Analysis

Reaction mixtures were analyzed by gas chromatography (GC) using an Agilent 6890N GC system equipped with a flame ionization detector (FID) and a capillary nonpolar column. Identification of the products was confirmed by GC-MS (Shimadzu QP2010). Details about analyses are given in Tables S1–S5 in the Supporting Information.

The conversion was calculated from the relative concentration data obtained by gas chromatography. Conversion (*X*) represents the percentage of starting material that has reacted. The calculation is shown in eq 1, where *A*_0_ denotes the relative concentration of the compound at the beginning of the reaction, and *A*_τ_ is the relative concentration at time τ:

1 $$X=\frac{A_{0}-A_{\tau }}{A_{0}}\cdot 100$$

Selectivity (*S*) was calculated from the same data set and expresses the proportion of the desired product relative to the total amount of all products formed. The calculation is shown in eq 2, where *A* is the relative concentration of the desired product, and Σ*P* is the sum of the relative concentrations of all products present in the reaction mixture:

2 $$S=\frac{A}{\sum P}\cdot 100$$

### Catalyst Characterization

The ARL 9400 XP sequential wave-dispersive X-ray spectrometer was used to perform X-ray fluorescence analysis (XRF). It is equipped with a 4GN Rh anode X-ray tube with a 50 μm thick Be end window. All spectral line intensities of the elements were measured in a vacuum by using the WinXRF program. The combination of generator–collimator–crystal-detector settings was optimized for 82 measured elements with a time of 6 s per element. The obtained intensities were processed by using the Uniquant 4 program without the need to measure standards. The analyzed powder samples were pressed into tablets with a thickness of 5 mm and a diameter of 40 mm without the use of a binder.

The specific surface area of all prepared catalysts was measured by nitrogen physisorption on a NOVA 2000e from Quantachrome Instruments Boynton Beach, FL, USA. Before the analysis, the samples were degassed at 300 °C for 2 h. Then, the 46-point adsorption/desorption isotherm was measured using nitrogen as an adsorbate at a constant temperature (77 K) of liquid nitrogen. The total pore volume was obtained from the isotherm at 0.986 P/P0. The micro- and mesopore size distribution and volume were evaluated by Density Functional Theory (DFT) and the Barrett–Joyner–Halenda method (BJH). The Brunauer–Emmett–Teller (BET) method was used for determination of the surface area of the samples. The surface area was calculated using the BET equation in the linear range of 0.05–0.30 P/P0.

Temperature-programmed desorption (TPD) of pyridine was performed to measure the acidity of the material (AutoChem III Micromeritics). A thermal conductivity detector (TCD) and quadrupole mass spectrometer (MKS Cirrus 2 Analyzer, MKS Instruments) with a capillary coupling system were used for desorbed pyridine detection. A catalyst sample (0.1 g) was placed in a U-shaped quartz U-shaped tube. Prior to adsorption of pyridine, the catalyst was heated under a helium flow (25 mL/min) up to 550 °C and kept at 550 °C for 120 min to remove impurities from the sample and clean the material surface. The sample was cooled to 100 °C and kept under pyridine–helium flow (vapor generator 40 °C) for 30 min until adsorption saturation. Then, the sample was flushed with helium for 60 min to remove physisorbed pyridine. Afterward, the linear temperature program (10 °C/min) was started at a temperature of 100 °C and the sample was heated to a temperature of 550 °C. The amount of desorbed pyridine was determined by calibration of the intensity of the 79 amu MS response (0.5 mL loop).

## Results and Discussion

The aim of this study was to develop an efficient one-pot synthesis of carvyl acetate from α-pinene oxide with a focus on maximizing the yield of carveol and carvyl acetate.

As is common in reactions involving isomerization, the acetalization of α-pinene oxide yielded a complex mixture of products. The identified compounds are illustrated in Figure 2. Several of these products—namely, FA, CA, PC, PCN, ECV, CVN, and CV—resulted from the isomerization of α-pinene oxide. Others, including PCAC, SAC, CAC, CVNAC, and CVAC, were formed through acetalization via distinct mechanistic pathways.

**Figure 2:**
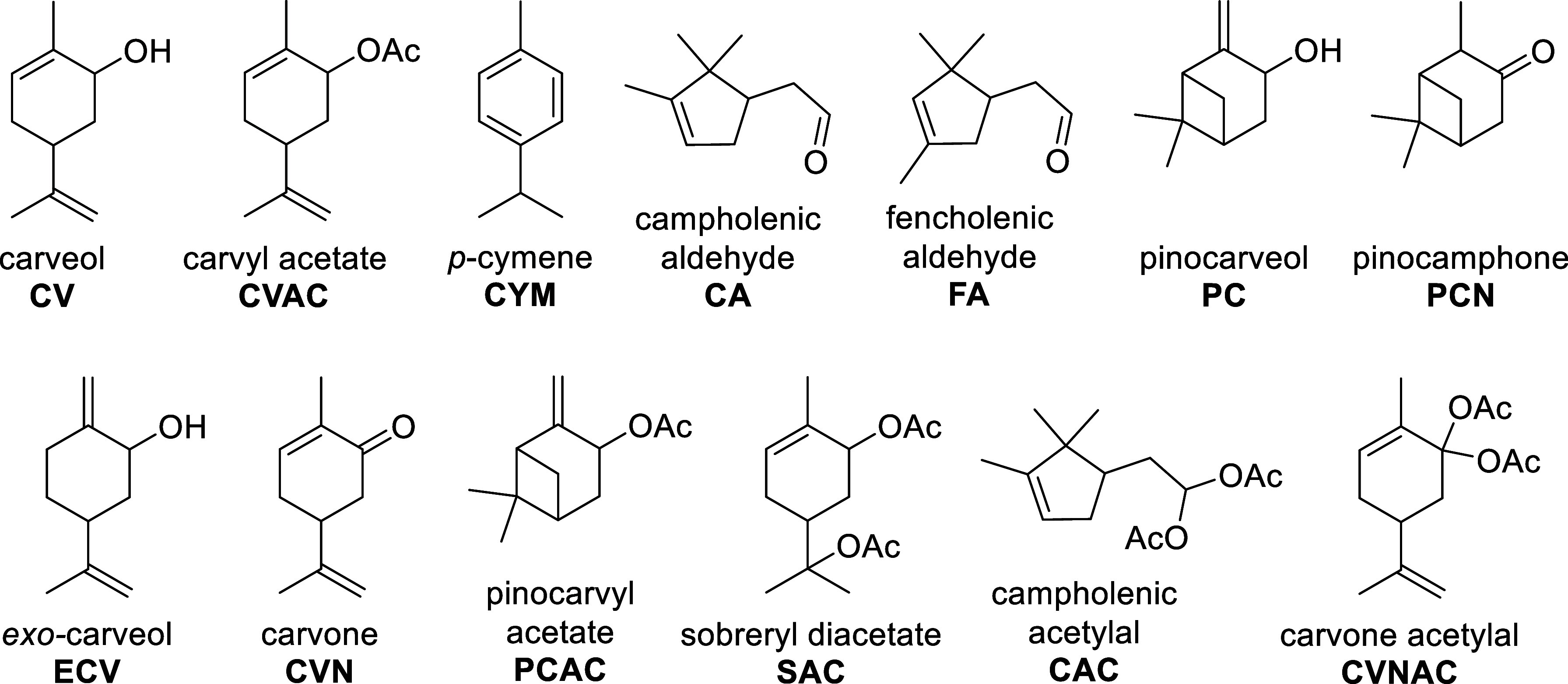
Structures of identified compounds in the reaction mixture.

Additionally, *p-*cymene (CYM) was detected in the reaction mixture, which is a typical byproduct frequently associated with the isomerization of pinene oxides. Among the various products, campholenic aldehyde (CA) was the most commonly observed. However, the primary objective of this study was to enhance the formation of carveol (CV) and, in particular, carvyl acetate (CVAC).

Given that acetic anhydride is typically used in excess and can also function as a solvent, we explored the possibility of conducting the acetalization of α-pinene oxide without the presence of any additional solvent. The molar ratio of α-pinene oxide to acetic anhydride was selected based on literature precedents.^18^

Total conversion of α-pinene oxide was achieved within 3 h using 5 wt % of catalyst (Figure S1). A lower catalyst loading confirmed the dependence of the reaction rate on the catalyst amount. However, regardless of the catalyst amount, only low concentrations of the desired products were obtained—3% carveol (CV) and 4% carvyl acetate (CVAC), as shown in Table 1.

**Table 1:** Selectivity to Products after 4 h of Reaction at Total Conversion^a^

		selectivity (%)							
catalyst amount (wt %)	conversion APO (%)	CV	CVAC	CA	FA	CYM	SAC	CAC	others
2	79.8	2.9	3.1	32.6	4.6	3.5	6.0	11.9	35.4
5	100	3.3	4.0	33.6	5.9	4.2	4.9	12.0	32.1

a Conditions: 1 g α-pinene oxide, 5.4 g Ac_2_O (molar ratio APO:Ac_2_O = 1:8), 50 °C, 2 and 5 wt % H-Beta 25.

The major product was campholenic aldehyde (CA, 33%), followed by campholenic acetylaldehyde (CAC), the product of CA acetylation. A significant portion of the reaction mixture consisted of unidentified products, which were present in sum in relatively high concentrations. The formation of unidentified products was attributed to the strongly acidic reaction environment created by the solid acid catalyst in the presence of acetic anhydride. Under these conditions, multiple parallel and consecutive reaction pathways may operate. Most of the identified products contain at least one carbon–carbon double bond, which makes them susceptible to further undesired transformations. These side reactions primarily include double-bond isomerization, oligomerization, dehydration, and acylation reactions.

In addition, mutual reactions between the initially formed products may occur, leading to more complex species. For example, campholenic aldehyde may undergo aldol condensation, while unsaturated compounds may be further acetylated. As a result, the reaction mixture may contain a highly complex distribution of products, including oligomeric, paraffinic, aromatic, and peracetylated species.

Under solvent-free conditions, the formation of peracetylated compounds is presumed to be particularly pronounced due to the high concentration of acetic anhydride.

Effect of Solvent

The low selectivity toward the desired product may be attributed to the strong influence of the solvent on the reaction outcome, which is typical for this type of transformation. Basic aprotic solvents are known to promote the formation of allylic alcohol structures,^14–16^ such as carveol and its acetate, which are the target compounds in this study.

To investigate this effect, we selected a set of solvents with varying polarity and donor properties: toluene (TOL), dimethylsulfoxide (DMSO), acetonitrile (AcN), *N,N,N′,N′*-tetramethylurea (TMU), *N,N-*dimethylacetamide (DMAc), and *N,N-*dimethylformamide (DMF). With the exception of toluene, all of these are basic aprotic solvents capable of stabilizing carbocation intermediates, thereby favoring the formation of our desired products. The selection was based on their ability to influence the reaction pathway through solvation effects and electronic stabilization.

In these experiments, reduced catalytic activity was observed in the presence of basic solvents. Although no direct interaction between the solvent and the catalyst was confirmed, the decrease in activity is likely due to partial neutralization or coordination of the acid sites by the basic solvent molecules. To compensate for this effect and to maintain sufficient catalytic performance, an increased catalyst loading (20 wt %) was applied.

A detailed discussion of the proposed reaction mechanism is provided later in the text. The selected physicochemical properties of the solvents used are summarized in Table S6 in the Supporting Information.

The influence of solvent properties on the conversion and selectivity of α-pinene oxide acetalization is summarized in Figure 5 and Table 2. These results illustrate the achieved conversion of α-pinene oxide and the selectivity toward the desired products—carveol and carvyl acetate.

**Table 2:** Selectivity to Products in the Given Solvents after 4 h^a^ of Reaction^b^

		selectivity (%)									
solvent	conversion APO (%)	CV	CVAC	CA	FA	CYM	CVN	SAC	CAC	CVNAC	others
DMF	83.5	43.1	3.8	30.2	11.9	1.3	–	–	–	–	9.7
DMAc	97.1	26.7	3.1	36.1	21.5	1.6	–	–	–	–	11.0
TMU	75.3	16.3	2.7	30.6	29.2	4.7	–	–	–	–	16.5
T^a^	100	6.4	2.4	43.2	8.5	6.0	–	6.1	3.5	–	23.9
AcN	51.5	4.6	1.9	42.2	7.0	2.2	–	–	1.9	–	42.1
DMSO	52.1	–	2.3	25.1	9.5	1.0	23.7	–	–	12.6	25.8

a Data for toluene after 15 min.

b Conditions: 1 g APO, molar ratio APO:Ac_2_O = 1:8, 4 mL solvent, 50 °C, 20 wt % H-Beta 25.

To evaluate the role of solvent characteristics, we examined several physicochemical parameters. Neither the donor number nor the relative permittivity (Figure 3) showed a clear correlation with conversion or selectivity. In contrast, solvent polarity, represented by the dipole moment, revealed a more pronounced trend: with the exception of the solvents with the lowest (toluene) and highest polarity (DMSO), both the conversion and the selectivity toward carveol increased with an increasing dipole moment (Figure 4). This observation may be attributed to enhanced stabilization of carbocationic intermediates, as discussed earlier. Interestingly, the selectivity toward the undesired campholenic aldehyde remained relatively constant across all solvents, regardless of their properties.

**Figure 3:**
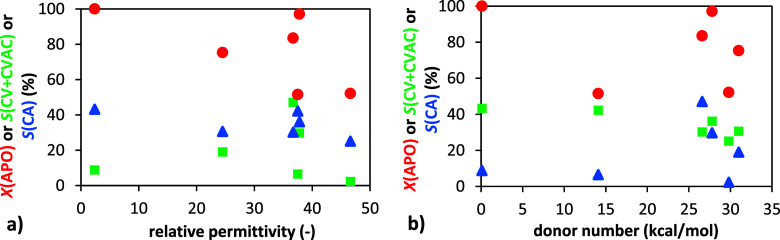
Dependence of α-pinene oxide conversion (●) and selectivity to carveol and carvyl acetate (■) and campholenic aldehyde (▲) on the donor number (a) and relative permittivity (b). Conditions: 1 g α-pinene oxide, molar ratio APO:Ac_2_O = 1:8, 4 mL of solvent, 50 °C, 20 wt % H-Beta 25.

**Figure 4:**
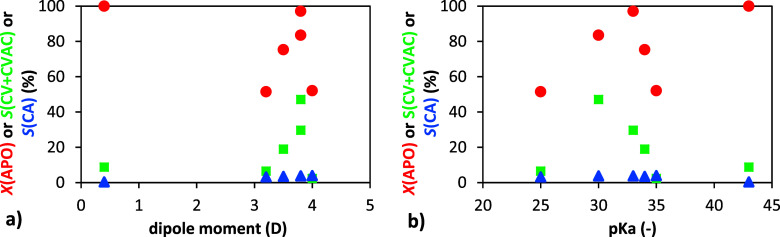
Dependence of α-pinene oxide conversion and selectivity to carveol and carvyl acetate on dipole moment (a) and p*K*_a_ (b). Conditions: 1 g α-pinene oxide, molar ratio APO:Ac_2_O = 1:8, 4 mL of solvent, 50 °C, 20 wt % H-Beta 25.

Further insights were obtained by analyzing the relationship between the solvent Brønsted acidity, expressed as p*K*_a_, and reaction performance (Figure 4). While the donor number reflects the Lewis basicity of the solvent, p*K*_a_ is associated with its Brønsted acidity. These two parameters describe different aspects of solvent behavior and do not necessarily correlate. Therefore, their influence on the reaction mechanism and product distribution may follow distinct trends.

For solvents with donor numbers up to approximately 30 kcal·mol^–1^ (i.e., acetonitrile, DMA, DMF, toluene), the conversion of α-pinene oxide increased with increasing p*K*_a_—that is, with decreasing Brønsted acidity. However, for solvents with donor numbers above ∼30 kcal·mol^–1^ (DMA, TMU, and DMSO), the trend reversed: conversion decreased with increasing p*K*_a_. This suggests that in highly basic solvents, the catalytic activity may be suppressed, likely due to interactions between the solvent and the acidic sites of the catalyst, which reduce its effective acidity (Figure 5).

When examining the selectivity toward carveol (Figure 5, excluding the extreme cases of toluene and acetonitrile), an unexpected negative correlation with p*K*_a_ was observed—higher p*K*_a_ values, which correspond to lower Brønsted acidity (and thus higher basicity), led to lower selectivity. This finding contrasts with the anticipated effect of increased carbocation stabilization in the more basic solvents. A possible explanation is that in highly basic environments, the interaction between the solvent and the catalyst may become too strong, reducing the effective acidity of the catalytic sites and thereby altering the reaction pathway unfavorably.

**Figure 5:**
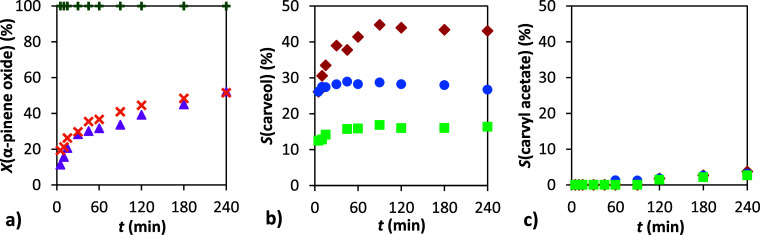
Dependence of conversion and selectivity on time: (a) conversion of α-pinene oxide, (b) selectivity to carveol, and (c) selectivity to carvyl acetate. Conditions: 1 g α-pinene oxide, molar ratio APO:Ac2O = 1:8, 4 mL of solvent: DMF (⧫), DMAc (●), TMU (■), DMSO (▲), AcN (×), T (**+**). 50 °C, 20 wt % H-Beta 25.

It is worth noting that DMSO,^19^ a solvent well-known for its ability to stabilize carbocations, exhibited unique behavior in this system. In addition to the expected products, carvone was detected in the reaction mixture, indicating a more complex reaction mechanism compared to that of the other solvents studied. It might be attributed to Albright–Goldman oxidation.^19^ We also expected that DMF and DMAc would help with the acetalization of carveol because both these solvents are known to activate the acetic anhydride. However, acetalization under the chosen conditions did not proceed.

The choice of solvent played a crucial role in the formation of carveol and carvyl acetate. The use of a nonpolar solvent such as toluene did not lead to any enhancement in either conversion or selectivity. In contrast, the application of basic solvents resulted in increased selectivity toward carveol; however, a decrease in the conversion of α-pinene oxide was observed, most likely due to interactions with the acidic sites of the catalyst. This interaction is attributed to physisorption, as no changes in pore size were observed (see catalyst characterization).

The role of the basic solvent is most likely associated with facilitating the formation of stable tertiary carbocation A (Figure 6), which acts as the key intermediate in the conversion to carveol. In contrast, the formation of the side product campholenic aldehyde proceeds via unstable secondary carbocation B (Figure 6). Consequently, carveol formation is favored under thermodynamically controlled conditions, whereas campholenic aldehyde formation is promoted under kinetically controlled conditions. This behavior is consistent with previously reported studies investigating solvent effects on the isomerization of α-pinene oxide.^9–11,13,15^

**Figure 6:**
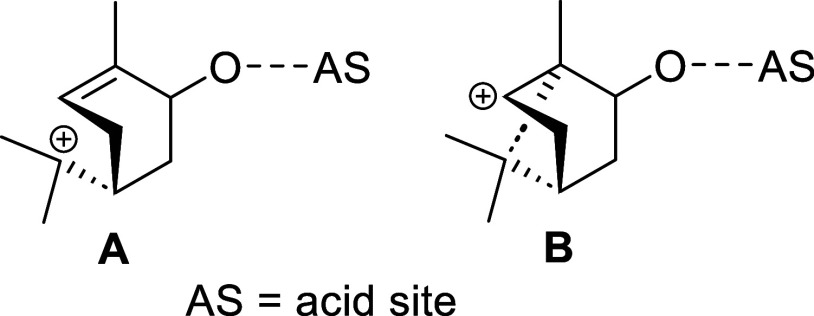
Carbocations in the isomerization system.

The ability of basic solvents to promote the formation of tertiary carbocation A can be primarily attributed to their high polarity, high relative permittivity, and elevated Lewis basicity. In this reaction system, the nature of the solvent functional group was also an important factor. Furthermore, potential interactions between the solvent and acetic anhydride, used as the acylating agent, had to be considered. For instance, in the case of DMSO, oxidative conditions were induced as a result of this interaction. Consequently, amidic solvents proved to be the most suitable under the applied conditions. In addition, basic solvents suppressed the formation of undesired byproducts by interacting with the acidic sites of the catalyst, thereby blocking these sites from acetic anhydride.

Contrary to our expectations based on previous experiments with the preparation of perillyl acetate,^17^ carveol was identified as a reaction intermediate in the current reaction pathway. The selectivity toward carveol formation remained nearly constant under the applied conditions. In contrast, the formation of carvyl acetate proceeded slowly, indicating that the chosen reaction parameters were not optimal for its efficient production. This observation prompted further investigation into alternative reaction conditions aimed at increasing the yield of carvyl acetate in the reaction mixture.

### Effect of Temperature

The effect of the temperature on the reaction course was investigated using DMF, which had previously shown the highest efficiency in the formation of carveol and carvyl acetate. The temperature range studied was 50–110 °C. As shown in Figure 7, temperatures above 50 °C led to rapid conversion of α-pinene oxide. Under the applied conditions, no significant differences in conversion were observed with increasing temperature. However, the temperature had a pronounced effect on product selectivity. At 50 °C, carveol was the predominant product, with only minimal formation of carvyl acetate, suggesting that lower temperatures favor accumulation of the intermediate. With an increase in temperature, the transformation of carveol to carvyl acetate became more pronounced. At 110 °C, carveol was rapidly consumed, and its concentration was already minimal within the first minute of the reaction, indicating fast acetylation. Nevertheless, a slight decrease in the carvyl acetate concentration was observed at prolonged reaction times, likely due to the formation of undesired secondary products.

**Figure 7:**
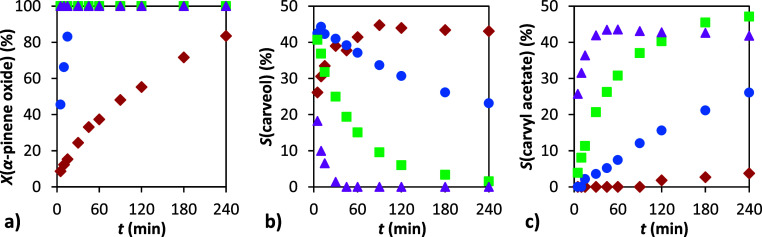
Dependence of conversion and selectivity on time: (a) conversion of α-pinene oxide, (b) selectivity to carveol, and (c) selectivity to carvyl acetate. Conditions: 1 g α-pinene oxide, molar ratio APO:Ac_2_O = 1:8, 4 mL DMF, 110 °C (▲), 90 °C (■), 70 °C (●), and 50 °C (⧫), 20 wt % H-Beta 25.

The highest selectivity toward carvyl acetate (47%) was achieved at 90 °C and remained stable under the applied conditions. This level of selectivity is comparable to values reported in the literature for similar catalytic systems. For example, Stekrova et al.^10^ investigated the isomerization of α-pinene oxide over nonmodified H-Beta 300 and Fe-modified H-Beta 25 zeolites, achieving selectivities of 42% and 32%, respectively, toward carveol at complete conversion of α-pinene oxide. However, these results were obtained under more severe reaction conditions (140 °C, DMAc as solvent, 25 wt % catalyst loading). In comparison, the selectivity achieved in this study was obtained at lower temperature and milder conditions, demonstrating the favorable performance of the H-Beta catalyst in the formation of carvyl acetate.

These findings confirm that carveol acts as a key intermediate in the reaction of α-pinene oxide with acetic anhydride and that the main pathway for carvyl acetate formation proceeds via the acetylation of carveol.

The composition of the reaction mixture after 4 h is summarized in Table 3. These results are consistent with the trends described above, confirming the highest concentration of carvyl acetate at 90 °C and the highest selectivity toward carveol at 50 °C. Interestingly, the combined selectivity toward both desired products—carveol and carvyl acetate—remained within a narrow range of 47–49% across all tested temperatures, with the exception of 110 °C, where a decrease was observed, likely due to the formation of undesired byproducts. A clear trend was also observed in the selectivity toward campholenic aldehyde, which decreased with increasing temperature. In parallel, the concentration of acetylated products increased—not only for the desired carvyl acetate but also for side products such as SAC and PCAC. This suggests that higher temperatures promote further acetylation reactions.

**Table 3:** Selectivity to Products at the Given Temperatures after 4 h of Reaction^a^

		selectivity (%)							
T (°C)	conversion APO (%)	CV	CVAC	CA	FA	CYM	SAC	PCAC	others
50	83.5	43.1	3.8	30.2	11.9	1.3	–	–	9.7
70	100	23.1	26.1	28.6	13.1	1.9	1.5	0.9	4.0
90	100	1.5	47.1	24.5	13.2	2.2	2.5	1.7	7.2
110	100	–	41.8	22.5	11.5	3.3	5.9	2.2	12.8

a Conditions: 1 g APO, molar ratio APO:Ac_2_O = 1:8, 4 mL DMF, 50, 70, 90, and 110 °C, 20 wt % H-Beta 25.

To determine the activation energy of the reaction, additional experiments were conducted at 100 °C, 90 °C, and 80 °C using a reduced catalyst loading (10 wt %). This adjustment was made to better capture the differences in the conversion behavior of α-pinene oxide over time. The time-dependent conversion profiles and corresponding selectivities are presented in Figure S2 in the Supporting Information. These data provide the basis for kinetic analysis and the subsequent calculation of activation energy.

### Effect of the Catalyst Amount

The amount of catalyst can significantly influence the reaction rate and the overall course of the reaction. After identifying 90 °C as the optimal temperature in terms of selectivity, we investigated the effect of catalyst loading on the reaction performance (Figure 8). The results confirmed that increasing the amount of catalyst led to a higher conversion rate of α-pinene oxide. In contrast, without the catalyst, the reaction did not proceed at all. Interestingly, the acetalization of the intermediate carveol to carvyl acetate appeared to be less sensitive to the catalyst amount. The time-dependent selectivity profiles showed only minor differences across the tested catalyst loadings. This suggests that the acetalization step, involving acetic anhydride, may proceed at least partially without catalytic assistance. As expected, the final composition of the reaction mixture (Table 4) showed only slight variations in the concentrations of carveol and carvyl acetate. The combined yield of both products, as well as the total amount of other byproducts, remained within the experimental error range. The higher amount of catalyst influence was tested at 50 °C (Table S7) and the same result was obtained: higher rate of α-pinene conversion and no influence on selectivity.

**Figure 8:**
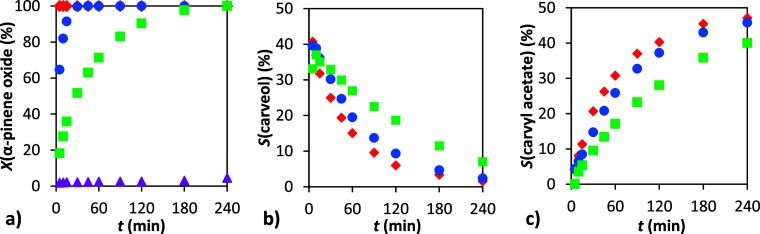
Dependence of conversion and selectivity on time: (a) conversion of α-pinene oxide, (b) selectivity to carveol, and (c) selectivity to carvyl acetate. Conditions: 1 g α-pinene oxide, molar ratio APO:Ac_2_O = 1:8, 4 mL DMF, 90 °C, 0 wt % (▲), 5 wt % (■), 10 wt % (●), and 20 wt % (⧫) H-Beta 25.

**Table 4:** Selectivity to Products at the Given Catalyst Amounts after 4 h of Reaction at Total Conversion^a^

	selectivity (%)							
*w*_kat_ (wt %)	CV	CVAC	CA	FA	CYM	SAC	PCAC	others
20	1.5	47.1	24.5	13.2	2.2	2.5	1.7	7.2
10	2.3	45.8	26.2	11.7	2.2	3.3	1.5	7.0
5	7.0	40.0	26.6	11.5	2.3	3.6	1.2	7.8

a Conditions: 1 g APO, molar ratio APO:Ac_2_O = 1:8, 4 mL DMF, 90 °C, 20, 10, and 5 wt % H-Beta 25.

### Effect of the Reactant Ratio at a Constant Amount of DMF

As previously discussed, the catalyst amount had only a minor effect on the transformation of carveol to carvyl acetate. Temperature was identified as the dominant factor influencing this step. In addition, we investigated the effect of the acetic anhydride concentration on the reaction outcome. From a mechanistic perspective, if the esterification followed Fischer’s principle, a stoichiometric (1:0.5) amount of acetic anhydride would theoretically suffice. However, in typical acylation reactions involving acetic anhydride, at least an equimolar amount is required, as one equivalent is consumed in forming acetic acid, which remains unreactive under the given conditions. To evaluate this, we tested three molar ratios of acetic anhydride to α-pinene oxide—1:1, 2:1, and 8:1—while keeping the volume of solvent (DMF) constant. The reactions were carried out at 90 °C using a relatively low catalyst loading of 5 wt %, which had previously proven sufficient. The reaction profiles and time-dependent selectivities are shown in Figure 9, and the final composition of the reaction mixtures is summarized in Table 5.

**Figure 9:**
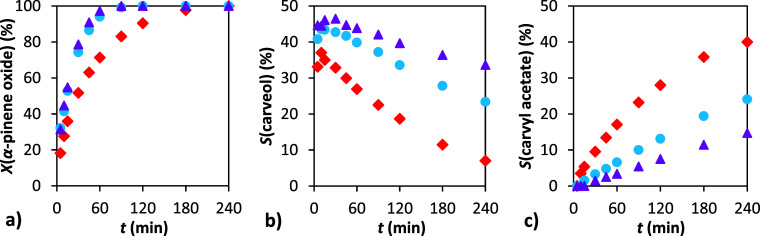
Dependence of conversion and selectivity on time: (a) conversion of α-pinene oxide, (b) selectivity to carveol, and (c) selectivity to carvyl acetate. Conditions: 1 g α-pinene oxide, molar ratio APO:Ac_2_O:DMF = 1:1:8 (▲), 1:2:8 (●), and 1:8:8 (⧫), 90 °C, 5 wt % H-Beta 25.

**Table 5:** Selectivity to Products at 3 Different Molar Ratios of APO to Ac_2_O after 4 h of Reaction at Total Conversion^a^

	selectivity (%)							
APO:Ac_2_O:DMF (Ac_2_O:DMF) (−)	CV	CVAC	CA	FA	CYM	SAC	PCAC	others
1:8:8 (1:1)	7.0	40.0	26.6	11.5	2.3	3.6	1.2	7.8
1:2:8 (1:4)	23.3	24.1	26.3	11.6	1.6	2.5	0.7	9.9
1:1:8 (1:8)	33.6	14.7	26.6	12.2	1.5	1.7	0.5	9.2

a Conditions: 1 g APO, molar ratio APO:Ac_2_O:DMF = 1:8:8, 1:2:8, 1:1:8, 90 °C, 5 wt % H-Beta 25.

As expected, the conversion of α-pinene oxide was only slightly affected by the amount of acetic anhydride. A minor decrease in the reaction rate was observed at the highest excess (8:1), likely due to dilution effects. More importantly, the selectivity toward the intermediate carveol remained largely unaffected by the acetic anhydride concentration. The lowest carveol selectivity was observed at the highest anhydride excess, which can be attributed to its rapid conversion to carvyl acetate. These results confirm that an excess of acetic anhydride plays a crucial role in the second step of the reaction—acetylation of carveol—enhancing the formation of the desired ester. Additionally, higher acetic anhydride concentrations led to increased formation of other acetylated byproducts (e.g., SAC, PCAC), consistent with trends observed in the temperature-dependent experiments.

## Effect of Solvent Volume

As previous experiments indicated a dilution effect influencing the first step of the reaction, we further investigated the impact of the solvent volume. While the presence of DMF was shown to be essential for the formation of carveol, we hypothesized that reducing its amount could improve catalyst performance by minimizing both dilution and potential interactions with the catalyst’s acidic sites. To test this hypothesis, we selected a molar ratio of α-pinene oxide to acetic anhydride of 1:2, which had previously shown no adverse effect on conversion. The reaction was carried out at 90 °C using three different volumes of DMF: 0.5, 2, and 4 mL.

The results, summarized in Figure 10, revealed that the solvent volume had no observable effect on the isomerization of α-pinene oxide. Similarly, the final composition of the reaction mixture (Table 6) remained largely unchanged when considering the combined selectivity to the two desired products. However, the second step of the reaction—acetylation of carveol—was clearly influenced by the amount of solvent. With increasing DMF volume, the rate of carveol conversion to carvyl acetate decreased, likely due to dilution effects. In this case, the catalyst appeared to play only a minor role, and the activation of acetic anhydride by DMF itself may require only a catalytic amount of solvent. Therefore, an excess of DMF did not enhance the reaction and may have even hindered the second step by reducing the effective concentration of reactive species.

**Figure 10:**
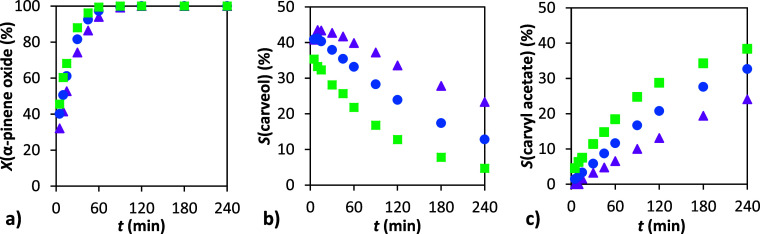
Dependence of conversion and selectivity on time: (a) conversion of α-pinene oxide, (b) selectivity to carveol, and (c) selectivity to carvyl acetate. Conditions: 1 g α-pinene oxide, molar ratio APO:Ac_2_O:DMF = 1:2:8 (▲, eq to 4 mL DMF), 1:2:4 (●, eq to 2 mL DMF), and 1:2:2 (■, eq to 0.5 mL DMF), 90 °C, 5 wt % H-Beta 25.

**Table 6:** Selectivity to Products at 3 Different Molar Ratios of APO to Ac_2_O after 4 h of Reaction at Total Conversion^a^

	selectivity (%)							
APO:Ac_2_O:DMF (Ac_2_O:DMF) (−)	CV	CVAC	CA	FA	CYM	SAC	PCAC	others
1:2:8 (1:4)	23.3	24.1	26.3	11.6	1.6	2.5	0.7	9.9
1:2:4 (1:2)	12.8	32.6	27.8	12.0	1.8	3.3	1.4	8.3
1:2:2 (1:1)	4.7	38.4	27.9	11.8	2.5	3.1	1.5	10.1

a Conditions: 1 g APO, molar ratio APO:Ac_2_O:DMF = 1:2:8, 1:2:4, and 1:2:2, 90 °C, 5 wt % H-Beta 25.

To further confirm that temperature is the key parameter influencing the second step of the reaction, and to better understand the unexpected absence of a dilution effect in previous experiments, we investigated the influence of solvent volume at a lower temperature (50 °C) using a catalyst loading of 20 wt %. The molar ratio of α-pinene oxide to acetic anhydride was set to 1:8. Under these conditions, the conversion of α-pinene oxide was strongly affected by the amount of DMF (Figure S3). As the solvent volume increased, the reaction rate—reflected by the conversion profile—decreased, likely due to the combined dilution effect of both the solvent and the large excess of acetic anhydride. The second step of the reaction, the acetylation of carveol, proceeded only to a very limited extent, supporting the conclusion that the temperature is the dominant factor driving this transformation. This observation motivated the calculation of the activation energy, which is discussed in the following sections.

Interestingly, the final composition of the reaction mixture was also influenced by the solvent volume (Table 7). At lower solvent volumes, a higher proportion of undesired byproducts—such as campholenic aldehyde, *p*-cymene, and others—was detected. These findings suggest the existence of an optimal ratio between acetic anhydride and DMF that balances reactivity and selectivity, leading to the most favorable reaction outcome.

**Table 7:** Selectivity Products at the Given Ratios after 4 h of Reaction^a^

		selectivity (%)							
APO:Ac_2_O:DMF (Ac_2_O:DMF) (−)	conversion APO (%)	CV	CVAC	CA	FA	CYM	SAC	PCAC	others
1:8:8 (1:1)	83.5	43.1	3.8	30.2	11.9	1.3	–	–	9.7
1:8:4 (2:1)	94.7	32.9	6.7	32.9	12.8	2.2	1.4	–	10.8
1:8:1 (8:1)	100	17.5	9.9	35.6	11.8	3.7	2.2	0.7	18.8

a Conditions: 1 g APO, molar ratio APO:Ac_2_O:DMF = 1:8:8, 1:8:4, and 1:8:1, 50 °C, 20 wt % H-Beta 25.

The previous experiments demonstrated that the ratio of acetic anhydride to DMF is a critical parameter that influences both steps of the reaction. An equimolar ratio of these two components was identified as optimal. Based on this finding, we investigated the effect of varying their combined excess relative to α-pinene oxide on the reaction course—specifically, the conversion of α-pinene oxide and the selectivity toward carveol and carvyl acetate (Figure 11). The reactions were carried out at 90 °C using 5 wt % catalyst.

**Figure 11:**
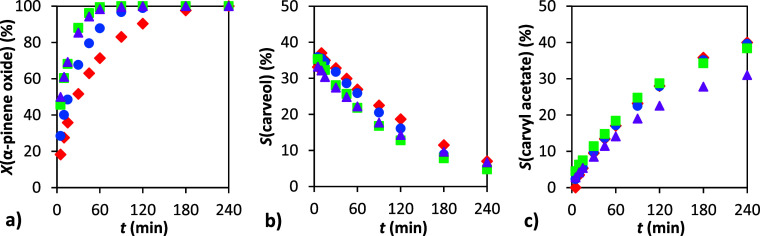
Dependence of conversion and selectivity on time: (a) conversion of α-pinene oxide, (b) selectivity to carveol, and (c) selectivity to carvyl acetate. Conditions: 1 g α-pinene oxide, molar ratio APO:Ac2O:DMF = 1:1:1 (▲), 1:2:2 (■), 1:4:4 (●), and 1:8:8 (⧫), 90 °C, 5 wt % H-Beta 25.

A clear dilution effect was observed in the conversion of α-pinene oxide when using higher excesses of both acetic anhydride and DMF. At a 1:1:1 and 1:2:2 ratio (α-pinene oxide:acetic anhydride:DMF), total conversion was achieved within 60 min. At a 1:4:4 ratio, complete conversion required 120 min, and at the highest excess (1:8:8), it was reached only after 240 min. These results confirm that increasing the total reaction volume slows the first step due to dilution of the reactants. In contrast, no significant dilution effect was observed in the second step—acetylation of carveol. Only a slight decrease in the carvyl acetate selectivity over time was noted in the equimolar system (1:1:1). Although the decline in carveol selectivity was comparable across all tested ratios, its reduced concentration in the equimolar system led to the formation of additional byproducts, such as campholenic aldehyde, fencholenic aldehyde, and others (Table 8).

**Table 8:** Selectivity to Products at 4 Different Molar Ratios of APO to Ac_2_O to DMF after 4 h of Reaction at Total Conversion^a^

	selectivity (%)							
APO:Ac_2_O:DMF (−)	CV	CVAC	CA	FA	CYM	SAC	PCAC	others
1:8:8	7.0	40.0	26.6	11.5	2.3	3.6	1.2	7.8
1:4:4	5.3	39.6	26.9	11.0	2.1	4.1	1.4	9.6
1:2:2	4.7	38.4	27.9	11.8	2.5	3.1	1.5	10.1
1:1:1	6.7	31.0	29.7	12.4	2.2	4.2	1.3	12.5

a Conditions: 1 g APO (ratio 1:8:8), 2 g APO (ratio 1:4:4 and 1:2:2) and 4 g APO (ratio 1:1:1), molar ratio APO:Ac_2_O:DMF = 1:8:8, 1:4:4, 1:2:2. 1:1:1, 90 °C, 5 wt % H-Beta 25.

The highest combined selectivity toward carveol and carvyl acetate was achieved at a 1:8:8 ratio. However, nearly identical selectivity was obtained at the 1:4:4 ratio, suggesting that this intermediate excess may offer a more efficient balance between the conversion rate and product distribution.

### Scale-up and Catalyst Reuse

In this part of the study, a catalyst loading of 10 wt % H-Beta 25 was used instead of 20 wt %. The lower catalyst amount allowed for clearer observation and monitoring of the reaction progress, whereas the higher loading led to complete α-pinene oxide conversion and the highest carvyl acetate yield within 5 min, limiting kinetic resolution.

Transferring reaction conditions from laboratory “micro-scale” to larger volumes can present significant challenges. Even small-scale scale-up experiments in the laboratory can provide valuable insights into the feasibility of potential industrial applications. To evaluate the scalability of the optimized reaction conditions, we increased the amount of α-pinene oxide 10-fold—from 1 to 10 g—while proportionally adjusting the quantities of all other reagents. The reaction was carried out under the same optimized conditions, and the results are presented in Figure 12 and Table S8.

**Figure 12:**
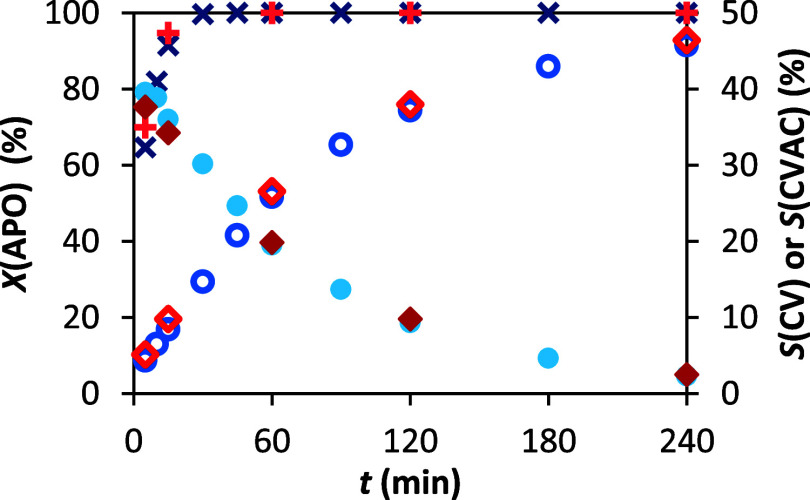
Dependence of conversion and selectivity on time. Conditions: 1 and 10 g α-pinene oxide, molar ratio APO:Ac_2_O:DMF = 1:8:8, 90 °C, 10 wt % H-Beta 25. Legend: conversion of APO (×), conversion of APO −10× (**+**), selectivity to CV (●), selectivity to CV −10× (⧫), selectivity to CVAC (○), and selectivity to CVAC −10× (◊).

As evident from both the figure and the table, the increased reaction scale had no observable effect on the reaction course. The isomerization of α-pinene oxide proceeded with similar kinetics, and the formation of carvyl acetate followed the same trend as in the “micro-scale” experiments. These findings suggest that the developed reaction system is robust and potentially suitable for further scale-up.

One of the key advantages of using heterogeneous catalysts is the possibility of their reuse. Given that zeolite beta is already employed in several industrially relevant processes, no major issues were anticipated regarding its recyclability in our system. Our experiments confirmed that a regeneration step—consisting of thorough washing followed by calcination—was necessary to remove residual organic compounds from the surface and pores of the zeolite. As shown in Table 9, and supported by the consistent achievement of full α-pinene oxide conversion within 15 min, no significant changes in product distribution were observed over three consecutive reaction cycles. A reduction in the initial amount of α-pinene oxide was applied in subsequent cycles to compensate for mechanical losses of the catalyst during handling. These losses occurred mainly during transfer and weighing steps and were estimated to be approximately 10–25% per cycle, resulting in a decrease from 100 mg of H-Beta 25 in the first cycle to about 50 mg in the third cycle. Despite these losses, the catalytic activity remained stable, confirming that H-Beta 25 is a suitable and robust catalyst for the preparation of carvyl acetate from α-pinene oxide under the tested conditions.

**Table 9:** Selectivity to Products When Using the Catalyst Repeatedly after 4 h of Reaction at Total Conversion^a^ ^b^

	selectivity (%)							
cycle (−)	CV	CVAC	CA	FA	CYM	SAC	PCAC	others
first (fresh)	2.3	45.8	26.2	11.7	2.2	3.3	1.5	7.0
second (F + W + C)	1.2	48.3	24.4	13.0	2.2	3.2	1.5	6.2
third (F + W + C)	2.0	48.7	25.4	13.5	2.0	2.7	1.4	4.3

a F = filtration, W = washing, C = calcination.

b Conditions: 1 g APO (1st and 2nd cycle), 0.5 g APO (3rd cycle), molar ratio APO:Ac_2_O:DMF = 1:8:8, 90 °C, 10 wt % H-Beta 25.

### Individual Reactions

We conducted individual reactions to improve our understanding of the behavior of the reaction system. The first isolated reaction, specifically the isomerization of α-pinene oxide, was examined under conditions identical to those used in the acetylation of α-pinene oxide, albeit in the absence of acetic anhydride. To compensate for the missing volume due to absence of acetic anhydride and thus to keep the concentration of α-pinene oxide consistent with previous experiments, toluene was used in the reaction mixture with volume corresponding to that of the same volume of acetic anhydride. Toluene was chosen instead of acetic anhydride to maintain a consistent initial concentration of α-pinene oxide while using a solvent that exerts minimal influence on the reaction system. As a nonpolar solvent, toluene is expected to have a negligible effect on reaction pathways, as discussed in the solvent effect section. Additionally, toluene is fully miscible with all other components of the reaction mixture and served as sample dilution prior to analysis.

The significant role of acetic anhydride in the reaction system during the initial step of the isomerization of α-pinene oxide is apparent (Figure 13). Within the first few minutes, the conversion of α-pinene oxide under conditions specific to isomerization was about 20%. In contrast, under conditions involving acetic anhydride, the conversion of α-pinene oxide exceeded 60% after 5 min. The total conversion was observed at 30 min under conditions involving acetic anhydride. In contrast, under conditions devoid of acetic anhydride, the conversion of α-pinene oxide was approximately 50% after 2 h. Acetic acid, which may be present in the system either from the possible interaction between acetic anhydride and the catalyst, resulting in its generation, or from its residual presence within the acetic anhydride, could further promote isomerization by providing additional acid sites within the reaction system.

**Figure 13:**
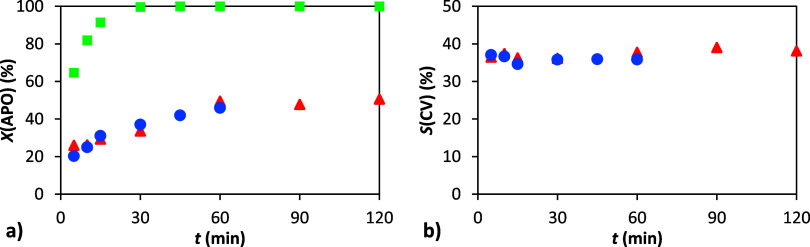
Dependence of conversion and selectivity on time: (a) conversion of α-pinene oxide; (b) selectivity to carveol. Conditions: 1 g α-pinene oxide, 5.4 g Ac_2_O (■) or 4.3 g toluene (●) or none (▲), 4 mL DMF (molar ratio APO:Ac_2_O:DMF = 1:8:8), 90 °C, 10 wt % H-Beta 25.

The use of DMF either alone or in conjunction with toluene within the reaction yielded comparable results concerning the conversion of α-pinene oxide and the selectivity for carveol. This suggests that the quantity of DMF, serving as a basic solvent, is more significantly influential than the initial concentration of α-pinene oxide (both reactions were conducted with identical amounts of DMF). However, the selectivity for carveol did not exceed 40%, which is comparable to the selectivity observed for the isomerization of α-pinene oxide over H-Beta 25 with DMAc as the solvent as reported by Stekrova et al.^10^

The subsequent acetylation of carveol was conducted under four distinct experimental conditions, each varying the quantity of catalyst utilized and the inclusion of DMF within the reaction mixture (Figure 14). The results indicate that the reaction proceeds in the absence of a catalyst, albeit at a slower rate compared to the catalyzed reactions in both cases (with and without the presence of DMF). In the first few minutes, the conversion of carveol in the absence of a catalyst was marginally below 40%. Without the presence of DMF, the conversion of carveol was nearly total after 2 h. The effect of the catalyst on the rate of the reaction (conversion of carveol to carvyl acetate) was examined by using 10 wt % catalyst loaded on a support. In the absence of the basic DMF solvent, the reaction proceeded almost immediately, with a total conversion achieved within 5 min. The presence of the basic solvent retarded the reaction, and the conversion of carveol was approximately 50% after 5 min and 90% after 60 min. The comparison between the noncatalyzed reaction devoid of basic solvent and the catalyzed reaction in the presence of a basic solvent presented a point of interest because both reactions yielded comparable results. This indicates that the presence of a catalyst, with a 10 wt % loading, counterbalances the effect of the basic solvent DMF, when utilized at a quantity of 4 mL, on the conversion of carveol. The selectivities observed in the studied reactions were similar within the range 75–95% and slightly increased in the first few minutes of the reactions.

**Figure 14:**
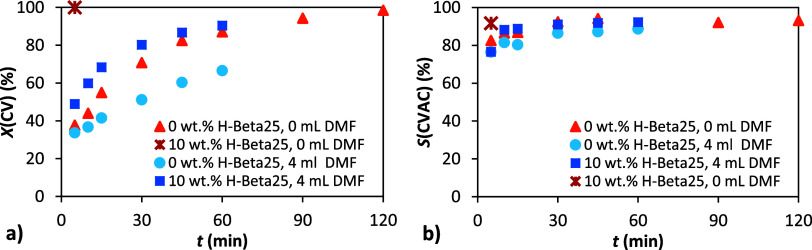
Dependence of conversion and selectivity on time: (a) conversion of carveol; (b) selectivity to carvyl acetate. Conditions: 0.5 g carveol, 5.4 g Ac_2_O, 0 or 4 mL DMF (molar ratio CV:Ac_2_O:DMF = 1:16:16), 90 °C, 0 and 10 wt % H-Beta 25.

### Mathematical Modeling

The reactor model was conceptualized as a batch model functioning within an intrinsic kinetic regime, and the mass balance for the organic components can be expressed as follows:

3 $$\frac{dn_{i}}{dt}=r_{i}\cdot m_{cat}\Leftrightarrow \frac{dc_{i}}{dt}=r_{i}\cdot \rho_{B}$$

where ρ_B_ is the catalyst bulk density, ρ_B_ = *m*_cat_/*V*_R_.

The model was constructed according to the simplified reaction scheme described in Figure 15.

**Figure 15:**
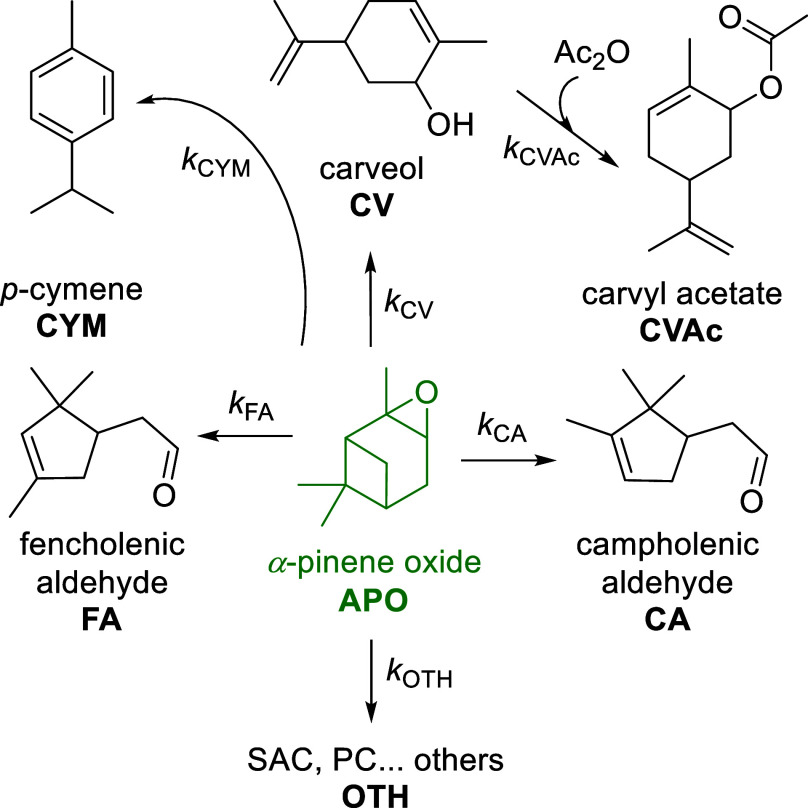
Reaction scheme for mathematical modeling.

The kinetic model was simplified by considering the key components of the reaction network: α-pinene oxide (APO) as the starting material, acetic anhydride (Ac_2_O) as the acetylation agent, carvone (CV) as the main intermediate, carvyl acetate (CVAc) as the final product, campholenic aldehyde (CA) and fencholenic aldehyde (FA) as significant side products, and *p*-cymene (CYM) as the dehydration product. All other identified minor byproducts, together with unidentified compounds, were grouped into a single lumped fraction denoted as OTH.

The isomerization of α-pinene oxide was described by first-order kinetics with respect to APO. This assumption is consistent with previously published kinetic studies on the acid-catalyzed isomerization of α-pinene oxide^14^ and reflects a reaction regime in which the rate is primarily controlled by the concentration of the reactant, while the number of active acid sites remains effectively constant.

The formation of CV, CA, FA, and CYM was assumed to occur via parallel reaction pathways originating from APO. The subsequent acetylation of CV to CVAc was modeled using second-order kinetics, accounting for the involvement of both CV and acetic anhydride in the rate-determining step. This kinetic order has been frequently reported for acid-catalyzed acetylation reactions and reflects the bimolecular nature of the process.

These assumptions allow a simplified yet physically meaningful description of the complex reaction network, capturing the dominant reaction pathways while keeping the number of kinetic parameters manageable. We obtained the following rate expressions (eqs 3–8):

4 $$r_{CV}=k_{CV}\cdot c_{APO}$$

5 $$r_{CVAc}=k_{CVAc}\cdot c_{CV}\cdot c_{{Ac}_{2}O}$$

6 $$r_{CA}=k_{CA}\cdot c_{APO}$$

7 $${r_{FA}=k}_{FA}\cdot c_{APO}$$

8 $$r_{CYM}=k_{CYM}\cdot c_{APO}$$

9 $${r_{OTH}=k}_{OTH}\cdot c_{APO}$$

To accurately determine activation energies, we consider the esteemed Arrhenius equation. Consequently, we present the following expressions as in eqs 9–14:

10 $$r_{CV}=k_{CV}^{\prime }\cdot \exp\frac{E_{CV}\cdot \left(T-T_{0}\right)}{R\cdot T\cdot T_{0}}c_{APO}$$

11 $$r_{CVAc}=k_{CVAc}^{\prime }\cdot \exp\frac{E_{CVAc}\cdot \left(T-T_{0}\right)}{R\cdot T\cdot T_{0}}\cdot c_{CV}\cdot c_{{Ac}_{2}O}$$

12 $$r_{CA}=k_{CA}^{\prime }\cdot \exp\frac{E_{CA}\cdot \left(T-T_{0}\right)}{R\cdot T\cdot T_{0}}\cdot c_{APO}$$

13 $${r_{FA}=k^{\prime }}_{FA}\cdot \exp\frac{E_{FA}\cdot \left(T-T_{0}\right)}{R\cdot T\cdot T_{0}}\cdot c_{APO}$$

14 $$r_{CYM}=k_{CYM}^{\prime }\cdot \exp\frac{E_{CYM}\cdot \left(T-T_{0}\right)}{R\cdot T\cdot T_{0}}\cdot c_{APO}$$

15 $${r_{OTH}=k^{\prime }}_{OTH}\cdot \exp\frac{E_{CYM}\cdot \left(T-T_{0}\right)}{R\cdot T\cdot T_{0}}\cdot c_{APO}$$

We obtained the changes in concentrations of compounds described through eqs 15–23 for this reaction system, all while adhering to the catalyst bulk density. The same catalyst bulk density (ρB) was used for all reaction steps, assuming that the acidic active sites of the catalyst participate similarly in the individual reactions. Consequently, variations in the catalyst amount proportionally affect the rates of all catalytic steps, while the relative contribution of individual pathways—and thus the selectivity—remains unchanged, in agreement with experimental observations.

16 $$dc_{APO}/dt=\left(-r_{CV}-r_{CA}-r_{FA}-r_{CYM}-r_{OTH}\right)\cdot \rho_{B}$$

17 $$dc_{CV}/dt=\left(r_{CV}-r_{CVAc}\right)\cdot \rho_{B}$$

18 $$dc_{CVAc}/dt=r_{CVAc}\cdot \rho_{B}$$

19 $$dc_{CA}/dt=r_{CA}\cdot \rho_{B}$$

20 $$dc_{FA}/dt=r_{FA}\cdot \rho_{B}$$

21 $${dc}_{CYM}/dt=r_{CYM}\cdot \rho_{B}$$

22 $$dc_{OTH}/dt=r_{OTH}\cdot \rho_{B}$$

23 $${dc}_{Ac_{2}O}/dt=-r_{CVAc}\cdot \rho_{B}$$

24 $$dc_{AcOH}/dt=r_{CVAc}\cdot \rho_{B}$$

### Kinetic evaluation

The reaction rate constants were systematically assessed for a specific reaction, with particular emphasis on quantifying the effects of the reaction conditions, such as the catalyst amount. In this context, we demonstrate only the values given for the formation of CV and CVAc. Subsequent values are presented alongside the fitted dependence of concentration on time within the Supporting Information (Figures S4–S19). Kinetics was performed in the software ERA 3.0 developed at UCT Prague. The software relies on the Gaines–Gaddy optimization algorithm.^20^ The Pascal programming language was used for the implementation of both the kinetic data and the model. Additionally, we assessed the ratio of the reaction rate constant values for carveol to carvyl acetate as a metric to quantify the transformation process from α-pinene oxide to carvyl acetate through carveol.

## Effect of Solvent

We evaluated only reactions with amidic solvents (DMF, DMAc, and TMU) because these had a significant influence on CV formation (Table 10). The highest reaction rate constant for the formation of carveol was observed with the use of DMAc as the solvent. Nevertheless, the best yield of carveol was achieved with DMF (Table 2). The reaction rate constant value for carvyl acetate is subject to a significant standard deviation error, exceeding 100%. This considerable error can be attributed to the notably low yield of carvyl acetate. The balanced formation of carvyl acetate, represented by the increased ratio of rate constants, follows the following order: TMU > DMF > DMAc. Thus, the formation of carvyl acetate was the best balanced for TMU. However, the relevance of these values is questionable due to the high standard deviation error associated with the rate constants for carvyl acetate.

**Table 10:** Values of Reaction Rate Constants for Carveol and Carvyl Acetate with Standard Deviation Error in %, and Ratio of Rate Constants for Carveol and Carvyl Acetate^a^

solvent	*k*_CV_ (L·g^–1^·h^–1^)	*e* (*k*_CV_) (%)	*k*_CVAc_ (L^2^·mol^–1^·g^–1^·h^–1^)	*e* (*k*_CVAc_) (%)	$\frac{k_{CV}}{k_{CVAc}}$ (mol/L)
DMF	0.010	6	0.00029	197	35
DMAc	0.036	15	0.00037	1461	97
TMU	0.005	21	0.00067	159	7

a Conditions: 1 g α-pinene oxide, molar ratio APO:Ac_2_O = 1:8, 4 mL of solvent: DMF, DMAc, and TMU, 50 °C, 20 wt % H-Beta 25.

Effect of Temperature

The evaluation of reaction rate constants was carried out for two catalyst amounts, specifically 20 and 10 wt %, revealing insightful contrasts and driving a deeper understanding of their catalytic potential with temperature. The values for 20 wt % H-Beta 25 are in Table 11 and for 10 wt % H-Beta 25 in Table 12.

**Table 11:** Values of Reaction Rate Constant for Carveol and Carvyl Acetate with Standard Deviation Error in %, and Ratio of Rate Constants of Carveol and Carvyl Acetate^a^

T (°C)	*k*_CV_ (L·g^–1^·h^–1^)	*e* (*k*_CV_) (%)	*k*_CVAc_ (L^2^·mol^–1^·g^–1^·h^–1^)	*e* (*k*_CVAc_) (%)	$\frac{k_{CV}}{k_{CVAc}}$ (mol/L)
50	0.010	6	0.0003	197	35
70	0.161	3	0.002	3	77
90	3.879	7	0.011	3	348

a Conditions: 1 g α-pinene oxide, molar ratio APO:Ac_2_O = 1:8, 4 mL DMF, 90 °C, 70 °C, and 50 °C, 20 wt % H-Beta 25.

**Table 12:** Values of Reaction Rate Constants for Carveol and Carvyl Acetate with Standard Deviation Error in %, and Ratio of Rate Constant for Carveol and Carvyl Acetate^a^

T (°C)	*k*_CV_ (L·g^–1^·h^–1^)	*e* (*k*_CV_) (%)	*k*_CVAc_ (L^2^·mol^–1^·g^–1^·h^–1^)	*e* (*k*_CVAc_) (%)	$\frac{k_{CV}}{k_{CVAc}}$ (mol/L)
80	0.124	7	0.006	8	19
90	0.516	5	0.018	4	28
100	0.750	5	0.033	4	23

a Conditions: 1 g α-pinene oxide, molar ratio APO:Ac_2_O = 1:8, 4 mL DMF, 100 °C, 90 °C, 80 °C, 10 wt % H-Beta 25.

The temperature has a significant influence on the kinetics. It is evident that temperature has a crucial role in supporting the formation of both substances, carveol, and carvyl acetate. For 90 °C, the reaction rate constant for carveol was observed to be 24 times greater than at 70 °C and 388 times greater than at 50 °C. Thus, we achieved an exponential increase of the reaction constant as a function of temperature $\left(k_{CV}={5\cdot 10^{-6}e}^{0.149\cdot T},R^{2}=0.9999\right)$ . The standard deviation error was minimal, ranging from 3% to 7%. The reaction rate constant for carvyl acetate formation did not increase as much as that for carveol. The standard deviation error was low (3%), except for 50 °C, where the error exceeded 100%. We also obtained an exponential increase of the reaction constant as a function of temperature $\left(k_{CVAc}={3\cdot 10^{-6}e}^{0.09\cdot T},R^{2}=0.9997\right)$ . The ratio of the reaction rate constants demonstrates that the temperature exerts a significant influence on the rapid formation of carveol. Although carvyl acetate is subsequently formed, its formation is not as favored as that of carveol. The best balance for the formation of carvyl acetate through carveol occurs at 50 °C.

The reaction rate constant values associated with a lower catalyst loading (10 wt % H-Beta 25) exhibited a more gradual behavior with increasing temperature, as indicated in Table 12, compared to those associated with a higher catalyst loading (20 wt % H-Beta 25, Table 11). Instead of the original temperature difference of 20 °C, a reduced differential of 10 °C was used. At 90 °C, the reaction rate constant value increased by a factor of 4.2 compared to that at 80 °C. Furthermore, at 100 °C, the constant was 1.5 times higher than at 90 °C and six times higher than at 80 °C. The standard deviation error was minimal, ranging between 5% and 7%. It can be observed that although the formation of carveol and carvyl acetate increased, the magnitude of the increase was not as significant under the conditions of lower catalyst loading in comparison to higher catalyst loading. The increase in the formation rate can be characterized for carveol (*k*_CV_ = 0.0313 · *T* – 2.3537, *R* ^2^ = 0.9792) and for carvyl acetate (*k*_CVAc_ = 0.0014 · *T* – 0.1025, *R* ^2^ = 0.9959). The formation of carvyl acetate from carveol is well-distributed across the temperature range (80–100 °C) with using 10 wt % H-Beta 25 opposite to the results from the conditions (50–90 °C, 20 wt % H-Beta 25).

### Effect of the Catalyst Amount

The quantity of the catalyst plays a crucial role. The values of reaction rate constant for carveol and carvyl acetate with standard deviation error in %, and the ratio of rate constant for carveol and carvyl acetate, are depicted in Table 13. For the minimum catalyst quantity examined (5 wt % H-Beta 25), the reaction rate constant for carveol was predictably the lowest. The value for 20 wt % H-Beta 25 increased 7.5 times to that of 10 wt % H-Beta 25 and 30.8 times compared to 5 wt % H-Beta 25. The standard deviation error was small, ranging between 5% and 7%. The reaction rate constant for carveol exhibited an exponential dependence $\left(k_{CV}=0.046\;e^{0.2246\cdot m_{cat}},R^{2}=0.9992\right)$ . Very surprisingly, the reaction rate constant for carvyl acetate formation achieved its maximum value when utilizing a 5 wt % H-Beta 25 loading. The reaction rate constant for carvyl acetate could be more accurately characterized by a logarithmic dependence (*k*_CVAc_ = −0.012·*ln m*_cat_ + 0.0472, *R* ^2^ = 0.9897). The loading of the catalyst primarily promoted the initial reaction, namely, the isomerization of α-pinene oxide, rather than the subsequent reaction, the acetylation of carveol. As a result, an increased catalyst concentration may promote the interaction with acetic anhydride, resulting in the formation of acetic acid. This acid provides additional acid sites for isomerization. On the other hand, acetic acid is a less effective agent for acetylation than acetic anhydride. The values for the ratio of reaction rate constants decreased in the order of 20 wt % > 10 wt % > 5 wt %. When 5 wt % H-Beta 25 was employed, carveol experienced less retention within the reaction system, resulting in the most effective conversion to carvyl acetate.

**Table 13:** Values of Reaction Rate Constant for Carveol and Carvyl Acetate with Standard Deviation Error in %, and Ratio of Rate Constants for Carveol and Carvyl Acetate^a^ ^b^

*w* (wt %)	*k*_CV_ (L·g^–1^·h^–1^)	*e* (*k*_CV_) (%)	*k*_CVAc_ (L^2^·mol^–1^·g^–1^·h^–1^)	*e* (*k*_CVAc_) (%)	$\frac{k_{CV}}{k_{CVAc}}$ (mol/L)
20	3.879	7	0.011	3	348
10	0.516	5	0.018	4	28
5	0.126	5	0.028	8	4

a Effect of the reactant ratio at a constant amount of DMF.

b Conditions: 1 g α-pinene oxide, molar ratio APO:Ac_2_O = 1:8, 4 mL DMF, 90 °C, 5, 10, and 20 wt % H-Beta 25.

We also evaluated the effect of the reactant ratio at a constant amount of DMF. The reaction rate constant values for carveol and carvyl acetate, along with their respective standard deviation errors expressed as percentages, and the ratio of the rate constants for carveol to carvyl acetate are presented in Table 14. The lowest value of the reaction rate constant for carveol was observed at the ratio APO:Ac_2_O:DMF = 1:8:8. Nevertheless, the rate constant value increased as the quantity of acetic anhydride decreased. On the other hand, the lowest value of the reaction rate constant for carvyl acetate was obtained for the ratio of APO:Ac_2_O:DMF = 1:1:8. An increased concentration of acetic anhydride inhibited the isomerization process while facilitating the acetylation reaction. As the amount of acetic anhydride decreased, the retention of carveol in the reaction system increased. Consequently, a higher proportion of acetic anhydride leads to a decrease in the initial concentration of α-pinene oxide available for isomerization while concurrently promoting the formation of carvyl acetate from carveol. Notwithstanding, its degradation due to interactions with the catalyst to acetic acid could engage in a reaction with carveol to form carvyl acetate.

**Table 14:** Values of Reaction Rate Constant for Carveol and Carvyl Acetate with Standard Deviation Error in %, and the Ratio of Rate Constants for Carveol and Carvyl Acetate^a^

APO:Ac_2_O:DMF (−)	*k*_CV_ (L·g^–1^·h^–1^)	*e* (*k*_CV_) (%)	*k*_CVAc_ (L^2^·mol^–1^·g^–1^·h^–1^)	*e* (*k*_CVAc_) (%)	$\frac{k_{CV}}{k_{CVAc}}$ (mol/L)
1:8:8	0.126	5	0.028	8	4
1:2:8	0.180	4	0.013	6	14
1:1:8	0.184	3	0.011	6	17

a Conditions: 1 g α-pinene oxide, molar ratio APO:Ac_2_O:DMF = 1:1:8, 1:2:8 and 1:8:8, 90 °C, 5 wt % H-Beta 25.

## Effect of Solvent Volume

Finally, we evaluated the effect of the solvent volume. The determined values of reaction rate constants and their ratios are depicted in Table 15. The highest value of the reaction rate constant for carveol was for the ratio APO:Ac_2_O:DMF = 1:2:8. The reaction rate constants for carvyl acetate exhibited a comparable range, spanning from 0.011 to 0.013 L^2^·mol^–1^·g^–1^·h^–1^. The ratios of the reaction rate constants exhibited considerable similarity; however, the ratio of APO:Ac_2_O:DMF at 1:2:2 demonstrated the most favorable equilibrium for the formation of carvyl acetate within the reaction system.

**Table 15:** Values of Reaction Rate Constants for Carveol and Carvyl Acetate with Standard Deviation Error in %, and Ratio of Rate Constants for Carveol and Carvyl Acetate^a^

APO:Ac_2_O:DMF (−)	*k*_CV_ (L·g^–1^·h^–1^)	*e* (*k*_CV_) (%)	*k*_CVAc_ (L^2^·mol^–1^·g^–1^·h^–1^)	*e* (*k*_CVAc_) (%)	$\frac{k_{CV}}{k_{CVAc}}$ (mol/L)
1:2:8	0.180	4	0.013	6	14
1:2:4	0.156	5	0.011	6	14
1:2:2	0.143	6	0.013	7	11

a Conditions: 1 g α-pinene oxide, molar ratio APO:Ac_2_O:DMF = 1:2:8, 1:2:4, and 1:2:2, 90 °C, 5 wt % H-Beta 25.

Based on the findings, it has been established that the Ac_2_O:DMF ratio of 1:1 yielded a highly favorable production of carvyl acetate. Additionally, these ratios were subject to further evaluation. The observed values, reflective of the reaction behavior for this specific case, are presented in Table 16. The best formation of carveol within the reaction system was achieved by utilizing an APO:Ac_2_O:DMF ratio of 1:8:8. Nonetheless, the other ratios (1:2:2, 1:4:4) were also appropriate for utilization, as the proportionality of the reaction rate constants was comparable.

**Table 16:** Values of Reaction Rate Constants for Carveol and Carvyl Acetate with Standard Deviation Error in %, and Ratios of Rate Constants for Carveol and Carvyl Acetate^a^

APO:Ac_2_O:DMF (−)	*k*_CV_ (L·g^–1^·h^–1^)	*e* (*k*_CV_) (%)	*k*_CVAc_ (L^2^·mol^–1^·g^–1^·h^–1^)	*e* (*k*_CVAc_) (%)	$\frac{k_{CV}}{k_{CVAc}}$ (mol/L)
1:8:8	0.126	5	0.028	8	4
1:4:4	0.130	5	0.021	7	6
1:2:2	0.143	6	0.013	7	11
1:1:1	0.088	7	0.009	9	9

a Conditions: 1 g APO (ratio 1:8:8), 2 g APO (ratio 1:4:4 and 1:2:2) and 4 g APO (ratio 1:1:1), molar ratio APO:Ac_2_O:DMF = 1:8:8, 1:4:4, 1:2:2. 1:1:1, 90 °C, 5 wt % H-Beta 25.

Activation Energy

The subsequent kinetic parameter assessed was the activation energy associated with the formation of carveol and carvyl acetate. These values are listed in Table 17. The activation energy values for carveol were observed to be in the range from 116 to 128 kJ·mol^–1^, accompanied by a small standard deviation error of 2–6%. The activation energy values for carvyl acetate were about 88 kJ·mol^–1^ with a low standard deviation error (3–7%). It is evident that the isomerization process had higher activation energy; nonetheless, an elevated temperature was also necessary to effectively achieve the acetylation step.

**Table 17:** Values of Activation Energy for Carveol and Carvyl Acetate with Standard Deviation Error in %^a^

conditions	*e a*_CV_ (kJ·mol^–1^)	*e* (*Ea*_CV_) (%)	*Ea*_CVAc_ (kJ·mol^–1^)	*e* (*Ea*_CVAc_) (%)
20 wt %, 70 °C	128	2	88	3
10 wt %, 90 °C	116	6	89	7
20 wt %, 90 °C	127	3	87	6

a Conditions: 1 g α-pinene oxide, molar ratio APO:Ac_2_O = 1:8, 4 mL DMF, 100 °C, 90 °C, 80 °C, 10 wt % H-Beta 25 and 90 °C, 70 °C, 50 °C, 20 wt % H-Beta 25.

### Catalyst Characterization

In order to gain deeper insight into possible changes in the catalyst induced by the reaction, a comprehensive characterization of the fresh and spent samples was performed. X-ray fluorescence (XRF) spectroscopy and nitrogen physisorption were applied to evaluate the potential variations in chemical composition and textural properties. Furthermore, the concentration of acidic sites was quantified by temperature-programmed desorption (TPD) using pyridine as a probe molecule.

The chemical compositions of the fresh catalyst (before the reaction) and the spent catalyst (after the reaction), as determined by XRF analysis, are summarized in Table S9 in the Supporting Information, which reports the major oxide constituents. The comparison reveals only negligible differences between the fresh and spent samples. Although the observed variations slightly exceed the absolute measurement uncertainty, they remain within a comparable range. These results indicate that the aluminosilicate framework of the catalyst remains structurally intact throughout the reaction.

The nitrogen adsorption–desorption isotherms of the fresh and spent catalysts are shown in Figure S19 in the Supporting Information. In comparison with the fresh material, the spent catalyst exhibits a lower amount of adsorbed nitrogen. Nevertheless, both samples display adsorption–desorption behavior typical of mesoporous materials, including a comparable shape of the isotherms and a similar reduction in the desorption hysteresis loop.

Textural properties derived from nitrogen physisorption measurements are summarized in Table S10 in the Supporting Information. The fresh H-Beta 25 catalyst exhibited a high specific surface area, which decreased markedly after the reaction to approximately 72% of its initial value. Pore volumes were primarily evaluated using the Density Functional Theory (DFT) method. The micropore volume decreased to 62% of its original value, whereas the mesopore volume was reduced to 91%. As a result, the relative mesoporosity of the spent catalyst increased by approximately 6.5%. The change in mesopore volume determined by the BJH method (90%) was in good agreement with the value obtained from the DFT analysis.

The observed decrease in specific surface area during the reaction can be attributed to physisorption and chemisorption processes or fouling by reaction-derived species, which are predominantly located within the micropores. This preferential blockage of micropores consequently led to an apparent increase in the mesoporosity of the material.

The pore size distributions of the fresh and spent catalysts were further analyzed. The adsorption–desorption isotherms were evaluated using the Barrett–Joyner–Halenda (BJH) method, and the pore size distributions were derived from the desorption branches of the isotherms. The corresponding pore size distribution profiles are presented in Figure S20 in the Supporting Information.

A slight decrease in the total pore volume was observed for the spent catalyst, while the pore diameters remained largely unchanged. These findings suggest that the aluminosilicate framework of the catalyst is largely preserved during the reaction. The observed reduction in pore volume may be associated with partial occupation and/or blockage of the pore system by species that are physisorbed or chemisorbed on the catalyst surface as well as by fouling deposits formed under reaction conditions.

The concentration of acidic sites of the catalyst was determined by temperature-programmed desorption (TPD) using pyridine as a probe molecule and was found to be 794 μmol_py_g^–1^_cat_. The TPD profile exhibited a single desorption maximum at 187 °C, which suggests that the H-Beta 25 catalyst predominantly contains weakly acidic sites.

The catalytic performance was further evaluated in terms of the turnover number (TON). The general definition of TON is given in eq 24; for the purposes of this study, this expression was modified as shown in eq 25.

25 $$TON=\frac{n_{CVAC}}{n_{acid\;sites}}$$

26 $$TON=\frac{\frac{m_{APO}}{M_{APO}}\cdot \frac{X_{APO}}{100}\cdot \frac{S_{CVAC}}{100}}{n_{py/g_{cat}}\cdot m_{cat}}$$

Under the reaction conditions affording the highest yield of carvyl acetate at 10 wt % H-Beta 25 loading, a TON of each cycle ranged around 39 and the total TON for three following cycles was approximately 120.

## Conclusions

The one-pot synthesis of carvyl acetate was systematically investigated with respect to choice of solvent, temperature, catalyst loading, reactant molar ratios, and solvent amount to obtain the highest yield of carvyl acetate. Significant outcomes for the synthesis of carveol were obtained by utilizing basic solvents, particularly amidic solvents such as DMF, DMAc, and TMU, at a moderate temperature of 50 °C. The best solvent was DMF. Nonetheless, the temperature of 50 °C was found to be insufficient for the efficient formation of carvyl acetate. A detailed examination of the effect of the temperature revealed that the optimal temperature for the reaction was 90 °C. The quantity of catalyst was more important in the rate of formation of carveol than in the rate of formation of carvyl acetate. The highest amount of catalyst (20 wt % H-Beta 25) facilitated the formation of carvyl acetate. Furthermore, increased quantities of acetic anhydride resulted in greater production of carvyl acetate. The notable ratio of acetic anhydride to DMF was determined to be 1:1. The best yield of carvyl acetate (47%) was achieved with the ratio of APO:Ac_2_O:DMF = 1:8:8. Individual reactions were also examined. In the case of α-pinene oxide isomerization, the additional presence of acetic acid has a pivotal role in influencing the reaction pathway. Acetylation of carveol occurred in the absence of a catalyst at 90 °C in both cases (with or without DMF present in the reaction mixture). The kinetic parameters, including the reaction rate constants and activation energy, were systematically evaluated. The activation energy associated with the formation of carveol was found to be higher than that required for the formation of carvyl acetate. This work can serve as a basic start for the industrial production of carvyl acetate directly from α-pinene oxide.

## Supplementary Material



## Notes

Detailed descriptions of analytical methods (GC-FID and GC-MS); additional data for solvent-free experiments; list of solvents and their properties; results on the influence of temperature, catalyst amount, and solvent volume on the reaction course; selectivity data for scale-up; kinetic curves of concentration vs time for all effects; and XRF composition of the catalyst and its surface characteristics from nitrogen physisorption (PDF)

## †

Current address: Department of Physical and Macromolecular chemistry, Faculty of Science, Charles University, Hlavova 8, 128 44 Praha 2, Czech Republic
