# Supplementary material for: One-Pot Synthesis of Carvyl Acetate from α‑Pinene Oxide under Catalysis by Zeolite H‑Beta 25
Source: ACS Omega. 2026 Mar 19;11(12):19454–69. doi: 10.1021/acsomega.5c12898 (PMC13044838; doi:10.1021/acsomega.5c12898)
Supplement: Supplementary file 1 [file ao5c12898_si_001.pdf]

One-pot synthesis of carvyl acetate from α-pinene oxide under catalysis by zeolite H-Beta 25

*Anna Rejzková, Marek Plachý, Eliška Vyskočilová**

Department of Organic Technology, University of Chemistry and Technology, Prague, Technická 5, 166 28 Prague 6

* Corresponding author: Eliška Vyskočilová, eliska.vyskocilova@vscht.cz

Analytical methods

Table S1: Temperature program of GC-FID

Temperature of the injector	250 °C
Initial temperature	90 °C
Hold time of the initial temperature	4 min
First temperature ramp	6 °C/min
Time of the first temperature ramp	10 min
Second temperature ramp	20 °C/min
Time of the second temperature ramp	5 min
End temperature	250 °C
Hold time of the end temperature	4 min
Temperature of the detector	250 °C

Table S2: Parameters of column for GC-FID

Column	HP-5
Length	30 m
Inner diameter	0.35 mm
Layer thickness	0.25 µm
Carrying gas	hydrogen
Pressure	51 kPa
Flow in column	4 ml/min

Table S3: Temperature program of GC-MS (GC part)

Initial temperature	80 °C
Hold of the initial temperature	5 min
Temperature ramp	10 °C/min
End temperature	250 °C
Hold time of the end temperature	5 min

Table S4: Temperature program of GC-MS (MS part)

MS detector	SQ
Interface temperature	250 °C
Temperature of the ion source	220 °C
End temperature	250 °C
Voltage (sensitivity)	0.9 kV

Table S5: Parameters of column for GC-MS

Column	DBH-5
Length	35 m
Inner diameter	0.2 mm
Layer thickness	0.35 µm
Carrying gas	helium
Pressure	198.2 kPa
Flow in column	1.11 mL/min

Additional results

Solvent-free experiments

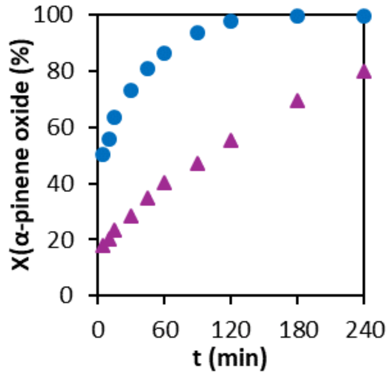

Figure S1: Dependence of *α*-pinene oxide conversion on time: 1 g *α*-pinene oxide, molar ratio APO:Ac_2O = 1:8, 50 °C, 2 wt% (▲) and 5 wt% (●) H-Beta 25.

Solvent effect

Table S6: List of solvents used, including their selected properties [1-3].

Solvent	Donor number (kcal mol^{-1})	Rel. permitivity (-)	Dipole moment (D)	pKa (-)
Acetonitrile	14.1	37.5	3.2	25
Dimethylacetamide	27.8	37.8	3.8	30-35
Dimethylformamide	26.6	36.7	3.8	30
Dimethlysulfoxide	29.8	46.6	4.0	35
Tetramethylurea	31.0	24.5	3.5	33-35
Toluene	0.1	2.38	0.4	43

Temperature effect

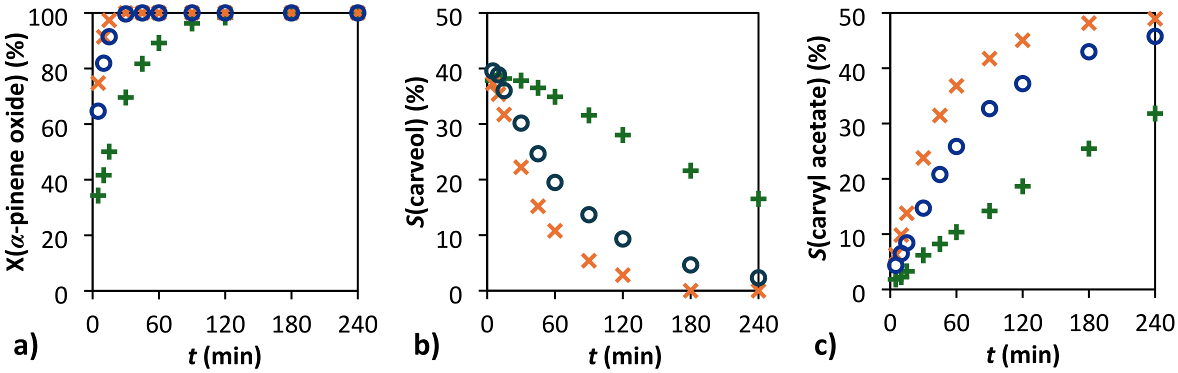

Figure S2: Dependence of conversion and selectivity on time: a) conversion of α-pinene oxide, b) selectivity to carveol, c) selectivity to carvyl acetate. Conditions: 1 g α-pinene oxide, molar ratio APO:Ac_2O= 1:8, 4 mL DMF, 100 °C (×), 90 °C (o), 80 °C (+), 10 wt.% H-Beta 25.

Catalyst amount effect

Table S7: Selectivity to products at given catalyst amounts after 4 h of reaction. Conditions: 1 g α-pinene oxide, molar ratio APO:Ac_2O = 1:8, 4 mL DMF, 50°C, 20, and 40 wt.% H-Beta 25.

w_{kat} (wt.%)	Conversion APO (%)	Selectivity (%)							
		CV	CVAC	CA	FA	CYM	SAC	PCAC	others
20	83.5	43.1	3.8	30.2	11.9	1.3	-	-	9.7
40	100	38.9	7.1	31.0	12.8	1.6	1.1	2.9	4.6

Solvent volume effect

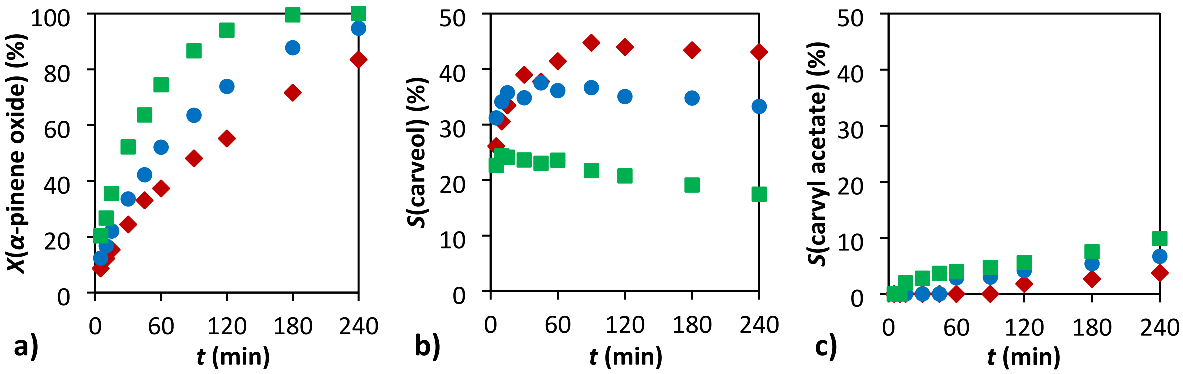

Figure S3: Dependence of conversion and selectivity on time: a) conversion of α-pinene oxide, b) selectivity to carveol, c) selectivity to carvyl acetate. Conditions: 1 g α-pinene oxide, molar ratio APO:Ac_2O:DMF = 1:8:1 (0.5 mL DMF) (■), 1:8:4 (2 mL DMF) (●) and 1:8:8 (4 mL DMF) (♦), 50 °C, 20 wt.% H-Beta 25.

Scale-up experiments

Table S8: Selectivity to products after 4 h at a 10-fold scale compared to experimental scale reactions after 4 h of reaction at total conversion. Conditions: 1 and 10 g APO, molar ratio APO:Ac_2O:DMF = 1:8:8, 90°C, 10 wt.% H-Beta25.

Scale (g APO)	Selectivity (%)							
	CV	CVAC	CA	FA	CYM	SAC	PCAC	others
1	2.3	45.8	26.2	11.7	2.2	3.3	1.5	7.0
10	2.5	46.4	25.9	12.0	2.2	3.2	0.3	7.5

Kinetic evaluation – concentration over time

Some dependencies of the concentration of substances over time were used several times for the comparison of many effects. We show only one as list, when they were used for the first time in the evaluation.

Legend: APO (*), CV(●), CVAc (●), CYM (■), CA (▲), FA (▼), OTH (▲)

Solvent effect

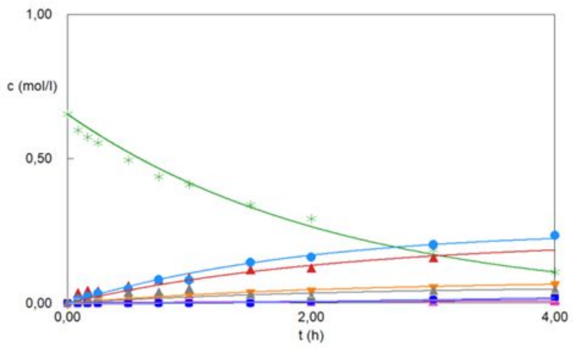

Figure S4: Dependence of concentration of substances on time. Conditions: 1 g *α*-pinene oxide, molar ratio APO:Ac_2O = 1:8, 4 mL DMF, 50°C, 20 wt.% H-Beta25.

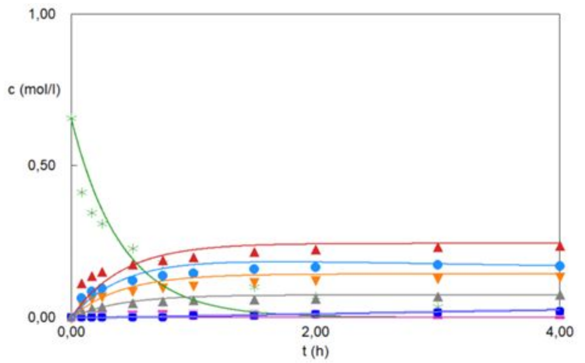

Figure S5: Dependence of concentration of substances on time. Conditions: 1 g α-pinene oxide, molar ratio APO:Ac_2O = 1:8, 4 mL DMAc, 50℃, 20 wt.% H-Beta25.

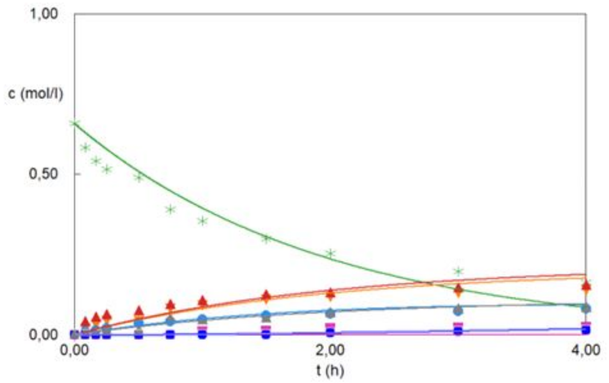

Figure S6: Dependence of concentration of substances on time. Conditions: 1 g α-pinene oxide, molar ratio APO:Ac_2O = 1:8, 4 mL TMU, 50℃, 20 wt.% H-Beta25.

Temperature effect

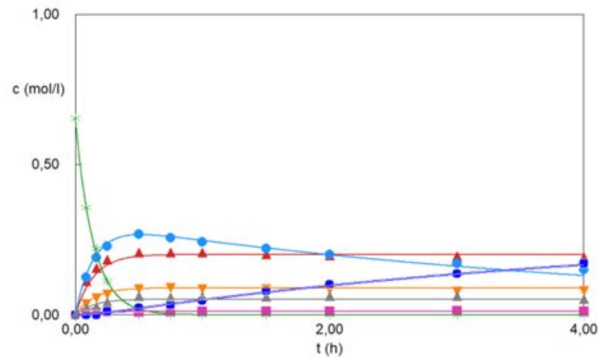

Figure S7: Dependence of concentration of substances on time. Conditions: 1 g α-pinene oxide, molar ratio APO:Ac_2O = 1:8, 4 mL DMF, 70℃, 20 wt.% H-Beta25.

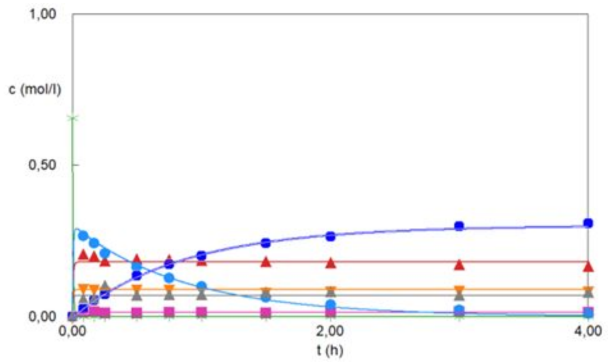

Figure S8: Dependence of concentration of substances on time. Conditions: 1 g α-pinene oxide, molar ratio APO:Ac_2O = 1:8, 4 mL DMF, 90°C, 20 wt.% H-Beta25.

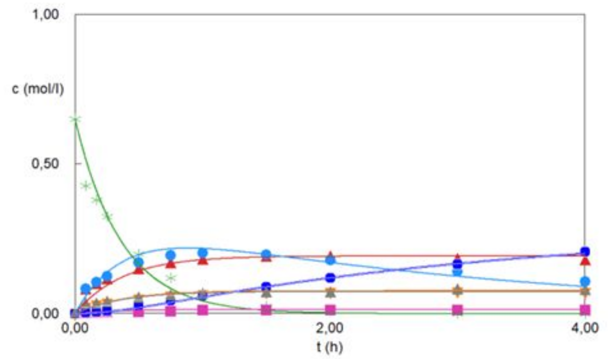

Figure S9: Dependence of concentration of substances on time. Conditions: 1 g α-pinene oxide, molar ratio APO:Ac_2O = 1:8, 4 mL DMF, 80°C, 10 wt.% H-Beta25.

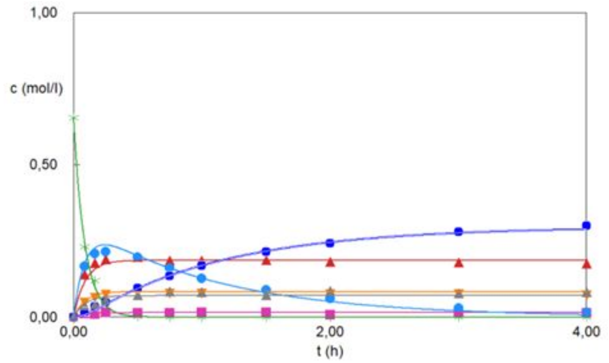

Figure S10: Dependence of concentration of substances on time. Conditions: 1 g α-pinene oxide, molar ratio APO:Ac_2O = 1:8, 4 mL DMF, 90°C, 10 wt.% H-Beta25.

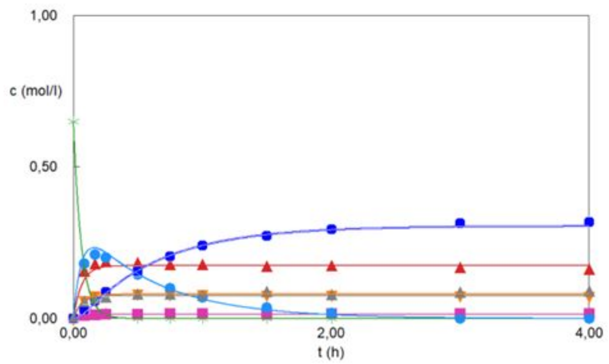

Figure S11: Dependence of concentration of substances on time. Conditions: 1 g *α*-pinene oxide, molar ratio APO:Ac_2O = 1:8, 4 mL DMF, 100℃, 10 wt.% H-Beta25.

Catalyst amount effect

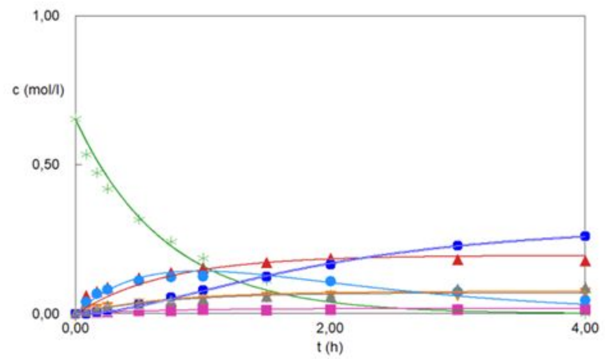

Figure S12: Dependence of concentration of substances on time. Conditions: 1 g *α*-pinene oxide, molar ratio APO:Ac_2O = 1:8, 4 mL DMF, 90℃, 50 wt.% H-Beta25.

Reactant ratio effect at a constant amount of DMF

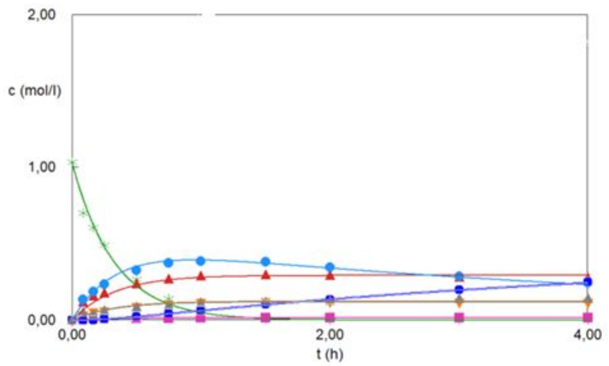

Figure S13: Dependence of concentration of substances on time. Conditions: 1 g α-pinene oxide, molar ratio APO:Ac_2O:DMF = 1:2:8, 90 °C, 5 wt.% H-Beta25.

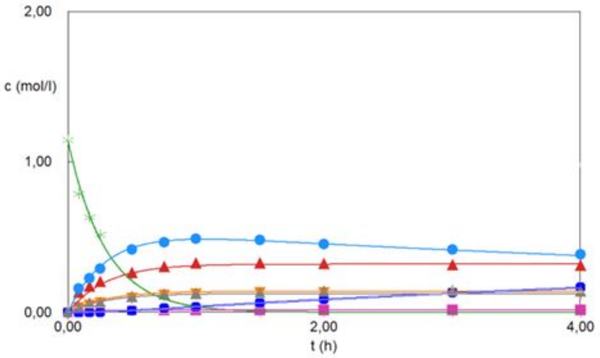

Figure S14: Dependence of concentration of substances on time. Conditions: 1 g α-pinene oxide, molar ratio APO:Ac_2O:DMF = 1:1:8, 90 °C, 5 wt.% H-Beta25.

Solvent volume effect

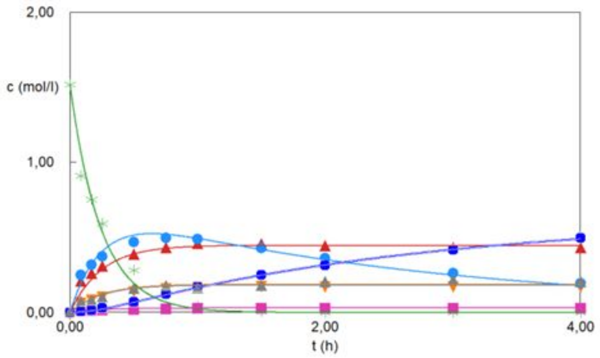

Figure S15: Dependence of concentration of substances on time. Conditions: 1 g α-pinene oxide, molar ratio APO:Ac_2O:DMF = 1:2:4, 90 °C, 5 wt.% H-Beta25.

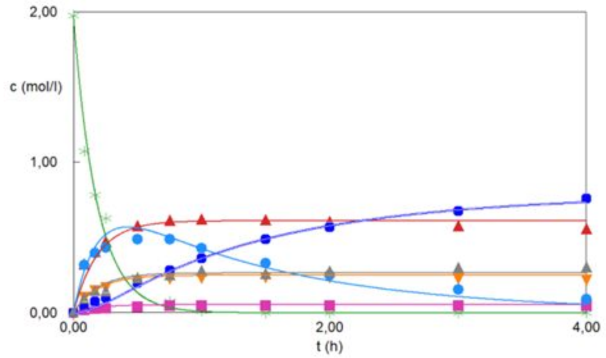

Figure S16: Dependence of concentration of substances on time. Conditions: 1 g α-pinene oxide, molar ratio APO:Ac_2O:DMF = 1:2:2, 90 °C, 5 wt.% H-Beta25.

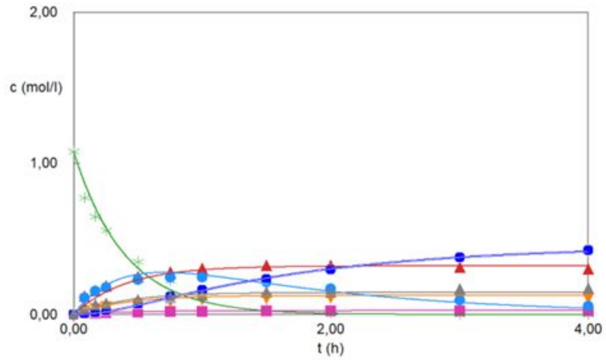

Figure S17: Dependence of concentration of substances on time. Conditions: 1 g α-pinene oxide, molar ratio APO:Ac_2O:DMF = 1:4:4, 90 °C, 5 wt.% H-Beta25.

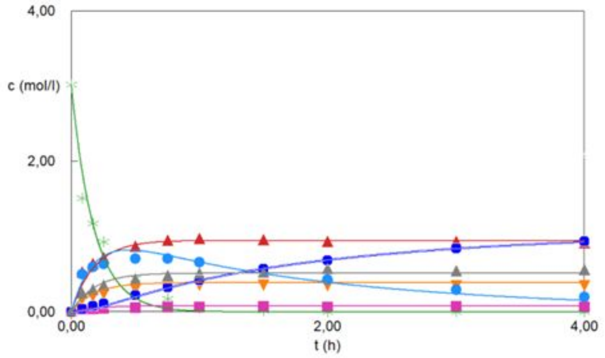

Figure S18: Dependence of concentration of substances on time. Conditions: 1 g α-pinene oxide, molar ratio APO:Ac2O:DMF = 1:1:1, 90 °C, 5 wt.% H-Beta25. Catalyst characterization

Table S9: Composition of main oxides in the catalyst with its absolute error

catalyst	SiO_2 (%)	abs. error (%)	Al_2O_3 (%)	abs. error (%)
fresh	94.0	0.1	5.7	0.07
spent	94.2	0.1	5.5	0.07

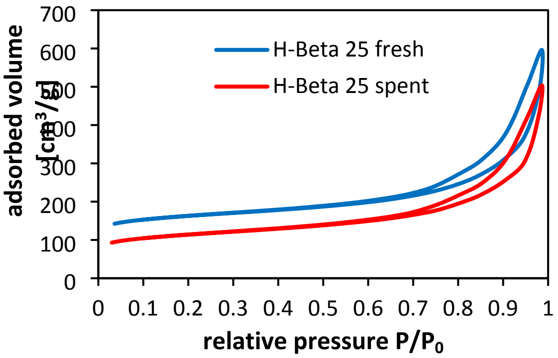

Figure S19: Nitrogen isotherms of fresh and spent H-Beta 25

Table S10: Specific surface area, volume of micropores and mesopores, mesoporosity

catalyst	S_{BET} (m^2/g)	DFT micropores (mL/g)	DFT mesopores (mL/g)	DFT mesoporosity (%)	BJH mesopores (mL/g)
fresh	523	0.18	0.55	75.4	0.59
spent	374	0.11	0.50	81.9	0.53

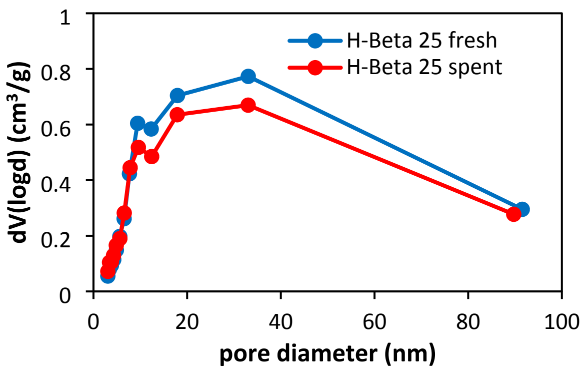

Figure S20: Pore size distribution

References

[1] Smallwood, I. M. Handbook of Organic Solvent Properties; Arnold, 1996.

[2] Stenutz, R. Tables for Organic Chemistry. 2025. http://www.stenutz.eu/chem/ (accessed Aug 09, 2025).

[3] Reich, H. Hans Reich's Collection. Bordwell pKa Table. 2025. https://organicchemistrydata.org/hansreich/resources/pka/ (accessed Aug 09, 2025).
